# MiXcan: a framework for cell-type-aware transcriptome-wide association studies with an application to breast cancer

**DOI:** 10.1038/s41467-023-35888-4

**Published:** 2023-01-23

**Authors:** Xiaoyu Song, Jiayi Ji, Joseph H. Rothstein, Stacey E. Alexeeff, Lori C. Sakoda, Adriana Sistig, Ninah Achacoso, Eric Jorgenson, Alice S. Whittemore, Robert J. Klein, Laurel A. Habel, Pei Wang, Weiva Sieh

**Affiliations:** 1 Tisch Cancer Institute, Icahn School of Medicine at Mount Sinai, New York, NY USA; 2 Department of Population Health Science and Policy, Icahn School of Medicine at Mount Sinai, New York, NY USA; 3 Department of Genetics and Genomic Sciences, Icahn School of Medicine at Mount Sinai, New York, NY USA; 4 Division of Research, Kaiser Permanente Northern California, Oakland, CA USA; 5 Regeneron Genetics Center, Tarrytown, NY USA; 6 Department of Epidemiology and Population Health, Stanford University School of Medicine, Stanford, CA USA; 7 Department of Biomedical Data Science, Stanford University School of Medicine, Stanford, CA USA

**Keywords:** Statistical methods, Gene expression, Breast cancer, Cancer genetics

## Abstract

Human bulk tissue samples comprise multiple cell types with diverse roles in disease etiology. Conventional transcriptome-wide association study approaches predict genetically regulated gene expression at the tissue level, without considering cell-type heterogeneity, and test associations of predicted tissue-level expression with disease. Here we develop MiXcan, a cell-type-aware transcriptome-wide association study approach that predicts cell-type-level expression, identifies disease-associated genes via combination of cell-type-level association signals for multiple cell types, and provides insight into the disease-critical cell type. As a proof of concept, we conducted cell-type-aware analyses of breast cancer in 58,648 women and identified 12 transcriptome-wide significant genes using MiXcan compared with only eight genes using conventional approaches. Importantly, MiXcan identified genes with distinct associations in mammary epithelial versus stromal cells, including three new breast cancer susceptibility genes. These findings demonstrate that cell-type-aware transcriptome-wide analyses can reveal new insights into the genetic and cellular etiology of breast cancer and other diseases.

## Abstract (web summary)

Conventional transcriptome-wide association study (TWAS) approaches predict genetically regulated gene expression at the tissue level. Here, the authors develop a framework for cell-type-aware TWAS that predicts cell-type level expression from genotype data and identifies disease-associated genes with cell-type-specific effects.

## Introduction

Transcriptome-wide association studies (TWAS) aim to identify genes that are associated with disease through their genetically regulated gene expression (GReX) levels^1,2^. Conventional TWAS approaches such as PrediXcan^1^ predict tissue-level GReX using models trained on transcriptomic and genomic data from bulk tissue samples, and test associations between the predicted tissue-level GReX and disease. By reducing the multiple testing burden from millions of variants to thousands of genes, TWAS can improve the power of genome-wide association studies (GWAS) while providing biological insights into the genes and regulatory mechanisms underlying disease. However, conventional TWAS approaches do not account for cell-type heterogeneity of bulk tissue samples, which can reduce the accuracy of GReX prediction models and obscure disease associations, particularly when the most mechanistically relevant cell type for the disease is a minor cell type in the tissue^3^.

Breast carcinoma is a common and highly heritable cancer that arises from epithelial cells, which line the ducts and lobules that produce milk during lactation^4,5^. Human mammary tissue has highly variable cell composition. Visualized on mammography, breast composition can range from extremely dense (light), reflecting a high proportion of fibroglandular tissue, to almost entirely fatty (dark), reflecting a high proportion of adipose tissue^6,7^. Whereas higher mammographic density is associated with increased risk of breast cancer, a higher amount of nondense fatty tissue is associated with decreased risk, indicating disparate roles of the different cellular components of mammary tissue in carcinogenesis^8–10^. Breast cancer susceptibility loci identified by prior GWAS^11–13^ and TWAS^14–16^ approaches that do not account for cell-type heterogeneity explain only a fraction of the familial relative risk. Disentangling the distinct effects of gene expression in mammary epithelial cells from other cell types through cell-type-aware analysis could lead to new gene discoveries and biological insights.

To our knowledge, no statistical methods currently exist for conducting cell-type-aware TWAS using GWAS data. Single-cell sorting and transcriptome profiling are costly, and large reference panels with both single-cell transcriptomic and genomic data are not yet widely available for training robust GReX prediction models. Recent studies of bulk tissue transcriptomic data have used computational estimates of cell-type enrichment, which are correlated with their proportions, to evaluate cell-type-specific effects. The Genotype-Tissue Expression (GTEx^17^) consortium estimated cell-type enrichment scores in bulk tissue samples using xCell^18^ and tested for interactions between genotype and xCell scores in linear regression models of gene expression to identify interaction expression quantitative trait loci (ieQTL)^19^. The breast was among the human tissues with the most ieQTLs, specifically involving mammary epithelial cells and adipocytes^19^, highlighting the potential for new methods that harness cell-type-specific genetic regulation of expression to improve the power of breast cancer TWAS. Methods that integrate bulk tissue data with single-cell reference profiles to estimate cell-type-level gene expression have also been proposed to study cell-type-specific disease associations^20,21^. However, these methods all require transcriptomic data from the disease-relevant tissue and cannot be applied to existing GWAS datasets to perform TWAS in large populations.

Here we present MiXcan, a new statistical framework for conducting cell-type-aware TWAS using GWAS data. MiXcan builds cell-type-level GReX prediction models through decomposition of bulk tissue data, identifies disease-associated genes via combination of signals from cell-type-level association analyses of multiple cell types, and provides insight into the cell type responsible for the disease association. We show that MiXcan improves the tissue-level GReX prediction accuracy compared with conventional approaches in an independent bulk-tissue validation set, and reliably predicts epithelial cell GReX in a single-nucleus RNA sequencing (snRNAseq) dataset. Simulation studies show that MiXcan controls the type I error, and provides higher power than conventional TWAS approaches when disease associations are driven by a minor cell type (e.g. mammary epithelial cells) rather than the predominant cell type in a tissue, or have opposite directions in different cell types. We apply MiXcan to conduct the first cell-type-aware TWAS of breast cancer risk in 31,716 cases and 26,932 controls, and report three new susceptibility genes (*ZNF703*, *TMEM245*, and *PSG4*) with evidence of distinct associations in mammary epithelial versus stromal cells that were not detected by prior TWAS nor GWAS. These findings provide a proof a concept that cell-type-aware TWAS can reveal new insights into the genetic and cellular etiology of breast cancer and other diseases.

## Results

### MiXcan framework

**Fig. 1:**
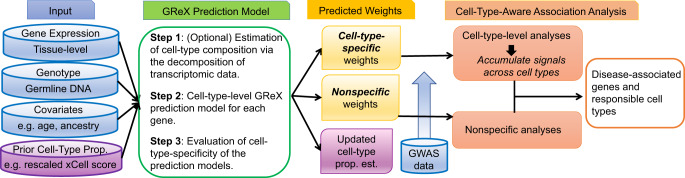
MiXcan framework. MiXcan estimates cell-type composition using transcriptomic data, builds cell-type-specific and nonspecific GReX prediction models, identifies disease-associated genes in any cell types, and provides insight into the cell type responsible for the disease association.

We developed the MiXcan framework for conducting cell-type-aware TWAS (Fig. 1). To build GReX prediction models, MiXcan requires specification of the cell type of interest and a prior estimate of its proportion in bulk tissue training samples with transcriptomic and genomic data. The cell type of interest for a given disease may be selected based on prior biologic knowledge, and its proportion estimated from the transcriptomic data using existing deconvolution methods and reference panels^21–23^. For cell types without large reference panels or direct proportion estimates, a cell-type enrichment score can be estimated from the bulk tissue transcriptomic data using xCell^18^. MiXcan can utilize xCell or other enrichment scores as a prior to estimate the cell-type proportion (see “Methods”). MiXcan then decomposes the bulk tissue gene expression level into its cell-type levels and uses joint penalized regression to model the association of genetic variants (SNPs) with gene expression for each cell type. The regression coefficients (SNP weights) are compared to determine whether the GReX prediction models for each gene are cell-type-specific (different weights in different cell types) or nonspecific (sames weights across cell types). Simulation studies (below) show that MiXcan prediction models are robust to misspecification of the cell-type proportion, which can result from inaccurate estimates^24–26^.

To conduct cell-type-aware TWAS, MiXcan uses the predicted GReX to test the following composite null and alternative hypotheses:

- *H*_0_: There is no association between the predicted GReX and the disease in any cell type.
- *H*_*A*_: There is an association between the predicted GReX and disease in at least one cell type.

Genes with cell-type-specific GReX prediction models are first associated with disease within each cell type, and then the signals are combined across cell types using the Cauchy-based *p*-value combination method^27^. Genes with nonspecific GReX prediction models are tested for their association with disease in one step. Significant associations are identified using an appropriate threshold to control the family-wise error rate (FWER) or false discovery rate (FDR). For significant genes with cell-type-specific GReX models, the cell-type-level results are compared to provide further insight into the cell type(s) likely to be responsible for the disease association.

While the MiXcan framework is general, its performance depends on the cell types under consideration and the available training data. As the number of cell types increases, the number of parameters increases and the accuracy of the model decreases. At present, given the limited sample sizes of transcriptomic and genomic datasets available for most human tissues through public repositories such as GTEx, it is practical to consider only two categories of cells using MiXcan and to focus on the cell type of greatest interest for the disease under investigation versus the other cell types. As breast carcinoma is known to arise from epithelial cells, we developed MiXcan epithelial and stromal (nonepithelial) cell models using bulk mammary tissue transcriptomic and genomic data available for 125 European ancestry (EA) women in GTEx v8.

### Prediction performance

The accuracy of MiXcan and PrediXcan GReX prediction models trained using GTEx v8 data for 125 mammary tissue samples from EA women was initially evaluated in an independent dataset of 103 tumor-adjacent normal mammary tissue samples from EA women in The Cancer Genome Atlas (TCGA). MiXcan estimates of the epithelial cell proportions were highly correlated with the xCell^18^ epithelial cell enrichment scores (used as a prior), with Pearson correlations (*r*) of 0.90 and 0.89 in normal mammary tissue samples from EA women in GTEx (N = 125) and TCGA (N = 103), respectively (Supplementary Fig. 1). However, MiXcan estimates of the epithelial cell proportion were more highly correlated with the expression levels of 126 genes included in the xCell epithelial cell gene signature (median *r* of 0.54 in GTEx and 0.60 in TCGA samples) than were the xCell enrichment scores themselves (median *r* of 0.36 in GTEx and 0.39 in TCGA samples) indicating that MiXcan can improve cell proportion estimation from its prior (Supplementary Fig. 1).

**Fig. 2:**
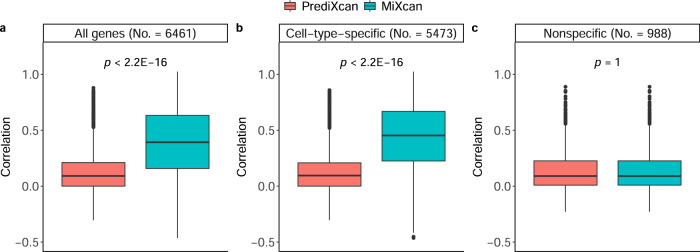
Validation of tissue-level GReX predictions in an independent bulk mammary tissue dataset. The correlation of tissue-level GReX predictions using MiXcan or PrediXcan with measured gene expression levels in adjacent normal mammary tissue samples from 103 European ancestry women with breast cancer in TCGA were computed for (**a**) all 6461 genes with MiXcan and PrediXcan models trained using mammary tissue samples from 125 European ancestry women in GTEx, (**b**) 5473 genes with cell-type-specific MiXcan models, and (**c**) 988 genes with nonspecific MiXcan models. Differences between the correlations for MiXcan and PrediXcan were compared using the two-sided Wilcoxon signed-rank test. Boxplot bounds show the lower, median, and upper quartiles; whisker lengths are 1.5 times the interquartile range; and points beyond the whiskers are outliers. Source data are provided as a Source Data file.

MiXcan estimated cell-type-specific prediction models for 5473 (84.7%) and nonspecific prediction models for 988 (15.3%) of 6461 genes that had mammary tissue-level prediction models available in PredictDB^28^ (Fig. 2). The tissue-level GReX was computed using MiXcan estimates of the cell-type proportion and predicted cell-type-level GReX values. The median correlation of predicted GReX and measured mammary tissue expression levels for the 6461 genes in the TCGA validation set was significantly higher for MiXcan compared with PrediXcan (median *r* of 0.41 vs. 0.10; *p* value < 2.2 × 10^−16^) models trained using the same dataset of 125 GTEx EA women. The prediction accuracy for the 5473 genes with cell-type-specific models in MiXcan was significantly better than PrediXcan (median *r* of 0.43 vs. 0.12; *p* < 2.2 × 10^−16^), whereas the prediction accuracy for the remaining 988 genes with nonspecific models in MiXcan was the same as PrediXcan (median *r* of 0.08 vs. 0.08; *p* value=1). These results indicate that allowing for cell-type-level GReX prediction models increases the prediction accuracy for genes with evidence of cell-specific genetic regulation, and does not decrease the prediction accuracy for other genes compared with standard approaches for predicting tissue-level GReX.

To examine potential sources of the gain in prediction accuracy, three additional approaches were compared with MiXcan and PrediXcan (Supplementary Fig. 2). The median correlation of predicted GReX with measured mammary tissue-level expression for all 6461 genes in the TCGA validation set was slightly higher for PredictDB (*r* = 0.12) elastic-net models trained using 337 GTEx EA men and women on the entire genome compared with PrediXcan (*r* = 0.10) trained using 125 EA women indicating modest gains from the inclusion of 212 EA men in the training dataset. Accounting for cell composition using penalized regression models including interactions of SNPs with the xCell epithelial cell score (xCell Interaction; *r* = 0.20) or MiXcan cell proportion (MiXcan_0_; *r* = 0.38) led to substantial gains in prediction accuracy. Symmetric estimation of cell-type-level prediction models employed in MiXcan (*r* = 0.41) further improved performance compared with standard interaction models that employ asymmetric penalization for the two cell types. Importantly, whereas standard interaction models require estimates of cell-type composition, which often are unavailable for the tissue of interest in GWAS of human diseases, MiXcan prediction models can be applied directly to GWAS genotype data to perform cell-type-aware TWAS.

**Fig. 3:**
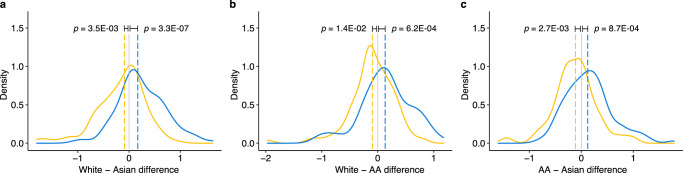
Validation of MiXcan epithelial cell GReX predictions using mammary epithelial cell snRNAseq data. Measured mammary epithelial cell snRNAseq levels for three GTEx women of White, Asian, and African-American (AA) ancestry were compared for six sets of 100 genes predicted to have the largest GReX differences in each pair of women. Distributions of the observed differences in the measured snRNAseq levels are shown for the 100 genes predicted to have the largest positive (blue) and negative (yellow) GReX differences for the (**a**) White − Asian, (**b**) White − AA, and (**c**) AA − Asian women. Dashed lines show the median of each distribution, and departures from zero were evaluated using the one-sided Wilcoxon signed-rank test. Source data are provided as a Source Data file.

Finally, to evaluate the prediction accuracy of MiXcan at the cell-type level, we compared the predicted epithelial cell GReX with measured mammary epithelial cell snRNAseq data available for three GTEx women of European, Asian, and African ancestry^29^. We found that genes (n=100) predicted to have the largest GReX differences based on the SNP genotypes in each pair of women also had significantly different measured snRNAseq levels in their mammary epithelial cells (*p* value range: 0.01 to 3.3 × 10^−7^), as expected (Fig. 3). The observed snRNAseq differences were significant despite the potentially poorer prediction accuracy of MiXcan models in women of Asian and African ancestry who were not represented in the training dataset. These snRNAseq results support the robustness of the cell-type level GReX predictions obtained using the MiXcan approach.

### Simulation studies

#### Type I error and power

To evaluate type I error and power of MiXcan association tests, datasets were simulated (see “Methods”) under a broad range of realistic settings for the associations of genetic variants with gene expression (SNP-Exp) and gene expression with disease (Exp-Disease). MiXcan predicted GReX with higher accuracy than PrediXcan in the presence of cell-type heterogeneity of SNP-Exp associations, while maintaining comparable accuracy in the absence of cell-specific effects (Supplementary Fig. 3), consistent with results in the independent TCGA validation dataset (Fig. 2).

**Fig. 4:**
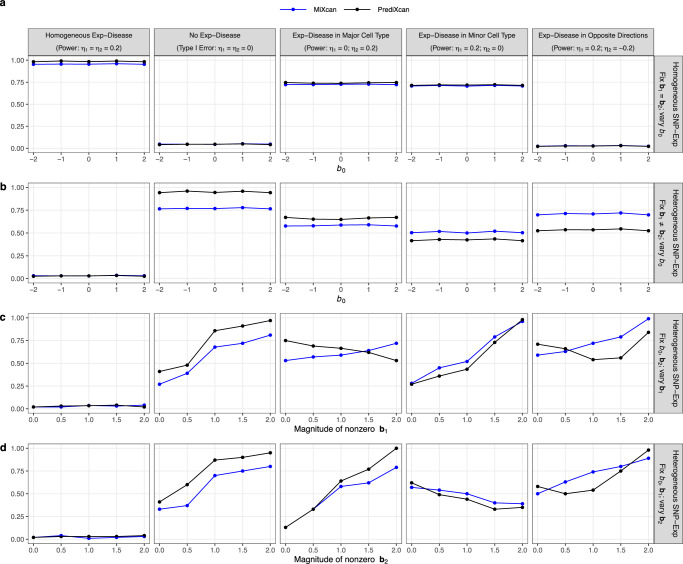
Simulation studies to evaluate the type I error and power of MiXcan and PrediXcan to detect associations of GReX with disease at the tissue level. Bulk tissue samples (*N* = 300) for training GReX prediction models and independent studies (*N* = 3000) for testing disease associations were simulated under a range of realistic data scenarios. Gene expression levels were modeled by *u* = *b*_0_ + **b**_1_*x* + *e*_*u*_ in the minor cell type, *v* = **b**_2_*x* + *e*_*v*_ in the major cell type, and *y* = *πu* + (1 − *π*)*v* at the tissue level, where *π* denotes the minor cell-type proportion, *b*_0_ denotes the mean difference of the gene expression levels in the two cell types, and **b**_1_ and **b**_2_ denote the weights for the association of SNPs X with gene expression levels in the minor and major cell types, respectively. The disease D was modeled by logit *P*(*D* = 1) = *η*_0_ + *η*_1_*u* + *η*_2_*v* where *η*_1_ and *η*_2_ denote the associations of the gene expression levels with disease in the two cell types, respectively. (**a**) Homogeneous SNP-Exp associations (**b**_1_ = **b**_2_) in the two cell types, varying the mean difference in gene expression levels between the two cell types (*b*_0_). Heterogeneous SNP-Exp associations (**b**_1_ ≠ **b**_2_) in the two cell types, varying the: (**b**) mean difference in gene expression levels between the two cell types (*b*_0_); (**c**) magnitude of the SNP-Exp association in the minor cell type (**b**_1_); and (**d**) magnitude of the SNP-Exp association in the major cell type (**b**_2_). Source data are provided as a Source Data file.

The type I error was well controlled for MiXcan and PrediXcan under all simulated data scenarios (Fig. 4 col. 1). When SNP-Exp associations were homogeneous in the two cell types (Fig. 4a), the power was similar for MiXcan and PrediXcan whether the Exp-Disease associations were homogeneous or heterogeneous across cell types. Differences in the mean gene expression level between the two cell types that were not determined by SNP-Exp associations (Fig. 4a, b) did not impact the power of MiXcan and PrediXcan indicating robustness to differential expression that is not regulated by genetic variants.

When SNP-Exp associations were heterogeneous in the two cell types (Fig. 4b–d), the relative power of MiXcan and PrediXcan depended on the mechanisms of the Exp-Disease and SNP-Exp associations. PrediXcan was generally more powerful than MiXcan when the Exp-Disease association was either homogeneous across cell types (Fig. 4 col. 2) or present only in the major cell type (Fig. 4 col. 3). However, MiXcan was generally more powerful than PrediXcan when the Exp-Disease association was present only in the minor cell type (Fig. 4 col. 4) or had opposite directions in the two cell types (Fig. 4 col. 5).

As the strength of the SNP-Exp association increased in the same cell type as the Exp-Disease association, the power increased for both PrediXcan and MiXcan (Fig. 4c col. 4; Fig. 4d col. 3). However, as the strength of the SNP-Exp association increased in a different cell type from the Exp-Disease association, the power decreased for PrediXcan but not MiXcan (Fig. 4c col. 3; Fig. 4d col. 4). When the Exp-Disease association had opposite directions in the two cell types, the power was U-shaped for PrediXcan but increased for MiXcan as the strength of the SNP-Exp association increased in either cell type (Fig. 4c-d col. 5). Similar patterns were observed when type I error and power were evaluated in relation to the expression heritability in the two cell types instead of the strength of SNP-Exp associations (Supplementary Fig. 4). These patterns show that different association signals in the two cell types can cancel each other out in PrediXcan, which averages their effects, but are aggregated across cell types in MiXcan thereby preserving power to detect associations due to the minor cell type or that differ across cell types.

In addition to providing valid tissue-level association tests, MiXcan provides information for each cell type separately. Simulation studies showed that the type I error was well controlled for MiXcan cell-type-level tests when no Exp-Disease association was present in any cell type (Supplementary Fig. 5 col. 1). When SNP-Exp associations were homogeneous across cell types (Supplementary Fig. 5a), MiXcan generally estimated nonspecific GReX prediction models which yield the same disease-association test results for all cell types. Thus, cell-type-level inferences can only be made in the heterogeneous SNP-Exp setting (Supplementary Fig. 5b–d), when MiXcan estimates cell-type-specific GReX prediction models. When the Exp-Disease association was present in both cell types in the same or opposite directions (Supplementary Fig. 5 cols. 2 & 5), the power of the cell-type-level tests was similar when the SNP-Exp associations had similar magnitude (regardless of direction) and increased as the magnitude of the SNP-Exp association increased. When the Exp-Disease association was present in only one cell type (Supplementary Fig. 5 cols. 3-4), the power was always highest in this cell type, but the association signal was shared to some degree with the uninvolved cell type. This correlation of the cell-type-level results arises from the joint estimation of the SNP weights for the cell-type-level GReX prediction models in MiXcan. Therefore, we recommend using the combined p-value for all cell types to make inferences regarding whether GReX is significantly associated with disease in any cell type in the tissue, and the cell-type-level results to compare the evidence that different cell types are involved for significant genes.

Finally, we evaluated the impact of the sample size of the training dataset on the type I error and power of MiXcan association tests in simulation studies (Supplementary Fig. 6). As the training dataset increased from 100 to 300 samples, the power of association studies with 3000 samples increased while the type I error remained well controlled. Prediction models trained using only 100-150 samples provided reasonable power for gene identification.

#### Performance under model misspecification

**Fig. 5:**
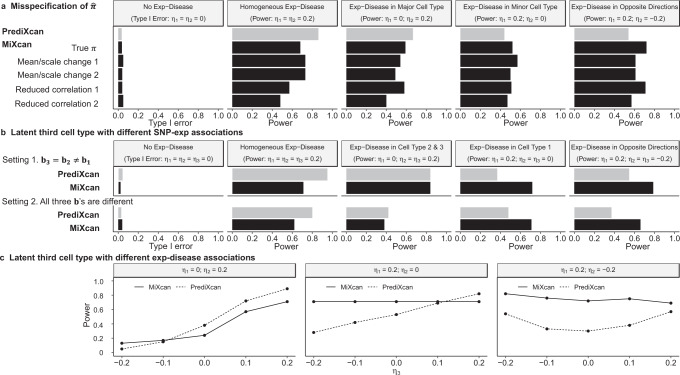
Simulation studies to assess the performance of MiXcan under model misspecification. **a** Type I error (column 1) and power (columns 2-5) when the estimated cell-type proportion $\hat{{{{{{{{\boldsymbol{\pi }}}}}}}}}$ used by MiXcan equals: the true ***π*** of 0.4; 0.8***π*** biasing the mean to 0.32 and changing the scale from (0, 1) to (0, 0.8) (mean/scale change 1); 0.7***π*** + 0.2 biasing the mean to 0.48 and changing the scale to (0.2, 1) (mean/scale change 2); *Beta*(50***π***, 50(1 − ***π***)) reducing the correlation with ***π*** to 0.9 (reduced correlation 1); and *Beta*(5.5***π***, 5.5(1 − ***π***)) reducing the correlation with ***π*** to 0.6 (reduced correlation 2). **b** Type I error (column 1) and power (columns 2–5) when a latent third cell type contributes to SNP-Exp associations. MiXcan was assumed to decompose the tissue into two components, cell type 1 (***π***_1_ = 40%) vs. a mixture of cell types 2 (***π***_2_ = 50%) and 3 (***π***_3_ = 10%). In Setting 1, the SNP-Exp associations were the same in cell types 3 and 2 and different in cell type 1 (**b**_3_ = **b**_2_ ≠ **b**_1_). In Setting 2, all three cell types had different SNP-Exp associations (**b**_3_ ≠ **b**_2_ ≠ **b**_1_). **c** Study power for a tissue with a latent third cell type that contributes to Exp-Disease associations under three heterogeneous Exp-Disease settings where *η*_1_ ≠ *η*_2_ and *η*_3_ varied from –0.2 to 0.2. Source data are provided as a Source Data file.

MiXcan decomposes bulk tissue expression levels into two components using an estimate of the cell-type proportion $\widehat{{{{{{{{\boldsymbol{\pi }}}}}}}}}$ for the cell of interest. First, we evaluated the performance of MiXcan under misspecification of $\widehat{{{{{{{{\boldsymbol{\pi }}}}}}}}}$ (Fig. 5a). In simulation studies using a broad range of biased and noisy estimates of $\widehat{{{{{{{{\boldsymbol{\pi }}}}}}}}}$, the type I error was consistently well controlled. The power of MiXcan also was generally maintained when $\widehat{{{{{{{{\boldsymbol{\pi }}}}}}}}}$ was misspecified, and compared favorably with PrediXcan when the Exp-Disease association was in the minor cell type or had opposite directions in the two cell types. Second, we evaluated the performance of MiXcan when a latent third cell type was present that had different SNP-Exp associations from the other cell types (Fig. 5b). We simulated a tissue with three cell types comprising 40%, 50% and 10% of the tissue, respectively, and assumed that MiXcan decomposed the tissue into cell type 1 versus a mixture of cell types 2 and 3. The type I error was consistently well controlled in the presence of a latent third cell type, and the power of MiXcan remained higher than PrediXcan when the Exp-Disease association was in cell type 1 (corresponding to the minor cell type in correctly specified models) or in opposite directions in cell types 1 vs. 2 and 3. The latent third cell type reduced the power of both PrediXcan and MiXcan when the Exp-Disease association was present in the most common cell type or homogeneous across all cell types. Third, we evaluated the impact of Exp-Disease associations in a latent third cell type on study power (Fig. 5c). Similar to the performance under correctly specified models, PrediXcan was more powerful mostly when Exp-Disease associations exist in cell type 2 (the most common cell type), and MiXcan was more powerful mostly when Exp-Disease associations exist in cell type 1 (the minor cell type of interest) or in opposite directions in cell types 1 and 2.

### Cell-type-aware TWAS of breast cancer

**Fig. 6:**
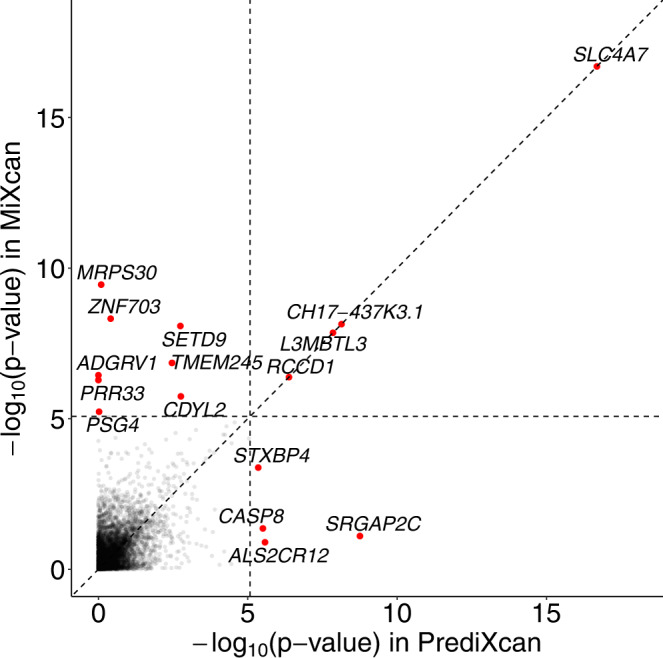
Transcriptome-wide association studies of breast cancer. MiXcan identified 12 genes and PrediXcan identified 8 genes that were significantly associated with breast cancer risk at *p* value < 7.7 × 10^−6^, applying a Bonferroni correction for the 6461 genes tested in 31,716 breast cancer cases and 26,932 controls of European ancestry from the DRIVE study. Source data are provided as a Source Data file.

As a proof of concept, we applied MiXcan to conduct the first cell-type-aware TWAS of breast cancer in a publicly available dataset of 58,648 EA women (31,716 cases and 26,932 controls) from the DRIVE GWAS (Discovery, Biology, and Risk of Inherited Variants in Breast Cancer) who were genotyped using the OncoArray^30^. Transcriptome-wide significance was determined using the Bonferroni-corrected threshold of 7.7 × 10^−6^ to account for the 6461 genes tested, and suggestive associations were determined using the Benjamini-Hochberg false discovery rate (FDR) of 0.10. MiXcan identified 12 significant genes (*p* value < 7.7 × 10^−6^) (Table 1, Fig. 6) and 82 suggestive genes (FDR < 0.10) (Supplementary Data 1) whose predicted GReX in mammary tissue were associated with breast cancer risk. In comparison, PrediXcan (trained using the same 125 EA female mammary tissue samples as MiXcan) identified only 8 significant genes (*p* value < 7.7 × 10^−6^), and 31 suggestive genes (FDR < 0.10). The inflation factor obtained using the BACON v1.26 Bayesian method to estimate the empirical null distribution was 1.01 for MiXcan and 1.03 for PrediXcan, indicating well-controlled type I error^31^.

**Table 1:** Genes significantly associated with breast cancer risk using the MiXcan or PrediXcan approaches in 58,648 women, and the corresponding S-PrediXcan and GWAS results in a larger sample of 228,951 women of European ancestry

Gene Name	Cytoband	MiXcan^a^							PrediXcan^b^			S-PrediXcan^c^			GWAS^d^ SNPs within 500 kb
		Epithelial			Stromal			Combined							
		Effect	SE	*P* value	Effect	SE	*P* value	*P* value	Effect	SE	*P* value	Effect	SE	*P* value	
Genes identified by both MiXcan and PrediXcan															
*CH17-437K3.1*^•^	1p11.2	–1.62	0.28	7.3E–09	–1.62	0.28	7.3E–09	7.3E–09	–1.62	0.28	7.3E–09	–0.56	0.05	3.1E–28***	rs11249433
*SLC4A7*^•^	3p24.1	2.30	0.27	2.0E–17	2.30	0.27	2.0E–17	2.0E–17	2.30	0.27	2.0E–17	1.11	0.09	3.5E–38***	rs4973768
*L3MBTL3*^•^	6q23.1	–0.12	0.02	1.4E–08	–0.12	0.02	1.4E–08	1.4E–08	–0.12	0.02	1.4E–08	–0.11	0.02	2.1E–11***	rs6569648
*RCCD1*^•^	15q26.1	–0.16	0.03	4.2E–07	–0.16	0.03	4.2E–07	4.2E–07	–0.16	0.03	4.2E–07	–0.16	0.02	3.5E–11***	rs2290203
Genes identified by MiXcan only															
*MRPS30*	5p12	3.00	0.67	6.8E–06	–4.82	0.75	1.8E–10	3.5E–10	–0.08	0.31	8.1E–01	0.50	0.04	3.3E–30***	rs10941679
*SETD9*	5q11.2	0.15	0.04	5.2E–04	–0.57	0.10	4.2E–09	8.4E–09	–0.07	0.02	1.8E–03	–0.10	0.02	1.9E–08***	rs62355902
*ADGRV1*	5q14.3	–0.27	0.05	3.6E–07	0.27	0.05	3.6E–07	3.6E–07	NA	NA	NA	–0.09	0.15	5.5E–01	rs10474352
*ZNF703*	8p11.23	–1.96	0.38	2.7E–07	1.59	0.27	2.4E–09	4.8E–09	0.07	0.08	3.9E–01	0.17	0.06	4.3E–03*	–
*TMEM245*	9q31.3	–0.64	0.12	7.0E–08	–0.30	0.10	2.2E–03	1.4E–07	–0.42	0.14	3.4E–03	0.04	0.07	5.4E–01	–
*PRR33*	11p15.5	–1.41	0.28	5.2E–07	1.41	0.28	5.2E–07	5.2E–07	NA	NA	NA	–1.39	0.14	1.4E–23***	rs3817198
*CDYL2*	16q23.2	–0.13	0.03	9.1E–07	–0.08	0.03	1.0E–03	1.8E–06	–2.67	0.85	1.7E–03	–0.10	0.04	3.7E–03*	rs13329835
*PSG4*	19q13.31	–0.16	0.04	3.0E–06	0.14	0.04	1.1E–04	5.9E–06	0.00	0.03	9.5E–01	0.01	0.01	5.3E–01	–
Genes identified by PrediXcan only															
*SRGAP2C*	1p11.2	–0.19	0.25	4.3E–01	–0.58	0.28	4.1E–02	7.8E–02	–0.65	0.11	1.8E–09	–0.56	0.05	1.2E–28***	rs11249433
*CASP8*	2q33.1	–0.65	0.30	2.7E–02	0.43	0.27	1.1E–01	4.4E–02	–0.13	0.03	3.1E–06	–0.11	0.02	3.1E–11***	rs1830298
*ALS2CR12*	2q33.1	–0.10	0.12	4.0E–01	0.40	0.22	7.0E–02	1.3E–01	0.18	0.04	2.6E–06	0.15	0.02	4.2E–12***	rs1830298
*STXBP4*	17q22	–0.06	0.07	4.1E–01	0.18	0.05	2.1E–04	4.2E–04	0.16	0.03	4.5E–06	0.32	0.03	3.4E–23***	rs2787486

^a^Genes with a MiXcan combined *p* value < 7.7 × 10^−6^ (0.05/6461 genes tested) aggregating across epithelial and stromal (non-epithelial) cell types were considered statistically significant. ^•^ denotes same model for epithelial and stromal cell types in MiXcan.

^b^Elastic net models were built using GTEx v8 mammary tissue data for 125 European ancestry women and all SNPs in the PredictDB database https://predictdb.org/. NA indicates that none of the SNPs evaluated were selected as predictors in the elastic net models for *ADGRV1* and *PRR33*. Genes with a PrediXcan *p* value < 7.7 × 10^−6^ were considered statistically significant.

^c^S-PrediXcan^33^ was used to test associations of predicted GReX with breast cancer risk using GWAS summary statistics for 122,977 cases and 105,974 controls^11^. *** denotes *p* value < 7.7 × 10^−6^ and * denotes *p* value < 0.05.

^d^Breast cancer SNPs reported in a GWAS of 122,977 breast cancer cases and 105,974 controls^11^.

Four significant genes (*CH17-437K3.1*, *SLC4A7*, *L3MBTL3*, and *RCCD1*) were identified by both MiXcan and PrediXcan (Table 1). MiXcan estimated nonspecific prediction models for these genes, yielding the same results as PrediXcan. All four genes were near (<500 kb) breast cancer SNPs previously identified by GWAS^11^, and two genes (*L3MBTL3* and *RCCD1*) also were reported by prior breast cancer TWAS (Supplementary Data 1)^14,15,32^. Follow-up analyses in a larger sample of 228,951 EA women (122,977 cases and 105,974 controls) in the Breast Cancer Association Consortium (BCAC) and DRIVE studies using GWAS summary statistics^11^ and S-PrediXcan^33^ models for mammary tissue confirmed that the tissue-level GReX for all four genes were significantly (*p* value < 7.7 × 10^−6^) associated with breast cancer risk.

Eight genes were identified by MiXcan but not PrediXcan (Table 1). MiXcan estimated cell-type-specific GReX prediction models for all eight of these genes (Supplementary Data 2). Six of these genes (*MRPS30*, *SETD9*, *ADGRV1*, *ZNF703*, *PRR33*, and *PSG4*) showed different directions of association with breast cancer in epithelial vs. stromal (nonepithelial) cells. In MiXcan the signals from the two cell types were aggregated, whereas in PrediXcan they canceled each other out reducing the tissue-level signal. Notably, for *ADGRV1* and *PRR33*, no SNPs in the training dataset were predictive of tissue-level GReX because of the mixture of the different cell-type effects, and consequently the PrediXcan association analysis could not be performed. Two genes (*TMEM245* and *CDYL2*) showed the same directions of association, with stronger effects in epithelial vs. stromal cells. These results indicate that MiXcan may be more powerful than PrediXcan in the presence of cell-type heterogeneity of GReX and when the disease association is present in a minor cell type, e.g. mammary epithelial cells, rather than the predominant cell type in the tissue.

Importantly, MiXcan uniquely identified three novel breast cancer susceptibility genes (*ZNF703*, *TMEM245*, and *PSG4*) that were not previously implicated by breast cancer GWAS^11–13^ nor TWAS^14–16,32^ (Table 1). For *ZNF703*, GReX was associated with increased breast cancer risk in stromal cells (*p* value=2.4 × 10^−9^) and decreased risk in epithelial cells (*p* value=2.7 × 10^−7^). Because adipocytes are the predominant stromal cell type in mammary tissue, we also performed follow-up analyses using the S-PrediXcan subcutaneous fat model and discovered a highly significant (*p* value=2.1 × 10^−20^) association of *ZNF703* with increased breast cancer risk in the four-fold larger BCAC/DRIVE dataset, consistent with the MiXcan stromal cell results in the DRIVE data only. For *TMEM245* and *PSG4* the signal was stronger in epithelial cells, which are a minor cell type in mammary tissue and may explain why their tissue-level GReX was not significantly associated with breast cancer risk. MiXcan also identified two breast cancer genes (*ADGRV1* and *CDYL2*) at previously reported GWAS loci^11^ that had different associations in epithelial and stromal cells and were not detected in prior TWAS.

Four genes (*SRGAP2C*, *CASP8*, *ALS2CR12*, and *STXBP4*) were identified by PrediXcan but not MiXcan (Table 1). There was high correlation between the predicted tissue-level GReX of *CH17-437K3.1* (also identified by MiXcan) and *SRGAP2C* at 1p11.2 (*r* = 0.95) and *CASP8* and *ALS2CR12* at 2q33.1 (*r* = –0.97) indicating that these associations may represent only two independent loci. All four genes identified by PrediXcan only were located near breast cancer SNPs previously identified by GWAS^11^, and three genes (*CASP8*, *ALS2CR12*, and *STXBP4*) also were reported by prior breast cancer TWAS (Supplementary Data 1)^15,32^. Associations of the mammary tissue-level GReX with breast cancer risk were found for all four genes in S-PrediXcan analyses of the BCAC/DRIVE data, as expected. MiXcan estimated cell-type-specific GReX prediction models for these four genes (Supplementary Data 2), and different associations with breast cancer risk in epithelial and stromal cells that did not reach statistical significance in part because of the larger number of model parameters compared with PrediXcan. However, the cell-type-specific MiXcan results for *STXBP4* suggest that stromal cells (estimated effect=0.18; *p* value = 2.1 × 10^−4^) may play a more important role than epithelial cells (estimated effect = –0.06; *p* value=0.41) in driving the positive association of tissue-level GReX with breast cancer risk.

Finally, we compared the TWAS results using MiXcan and PrediXcan with publicly available PredictDB^28^ elastic-net models trained using GTEx mammary tissue data for 212 EA men in addition to 125 EA women (Supplementary Fig. 7). There was substantial overlap of the genes detected by PrediXcan and PredictDB as expected, although PredictDB detected a larger number of genes perhaps because the larger training dataset enabled more accurate prediction models for genes that have similar expression patterns in male and female mammary tissue. However, the etiology and pathobiology of male breast cancer is distinct from female breast cancer^34^. Thus, prior TWAS of breast cancer in EA women^15,32^ also trained PrediXcan models using mammary tissue samples (n=67) from EA women only, as we did here using a larger number of EA women from GTEx. Cell-type-specific TWAS using MiXcan identified five genes (*ADGRV1*, *ZNF703*, *TMEM245*, *CDYL2*, and *PSG4*), including three novel breast cancer susceptibility genes, that were not identified by either PrediXcan or PredictDB.

## Discussion

MiXcan is a new statistical framework for conducting cell-type-aware TWAS using GWAS data. In contrast to standard TWAS methods, MiXcan builds cell-type-level prediction models for the genetically regulated component of gene expression and performs association tests taking into consideration the signals from multiple cell types. We have shown that MiXcan improves the prediction accuracy of GReX at both the tissue and cellular levels in independent validation datasets, and improves the power to detect disease associations that are driven by a minor cell type or are heterogeneous between cell types compared with standard approaches. We applied MiXcan to perform the first cell-type-aware TWAS of breast cancer risk and identified three new susceptibility genes (*ZNF703*, *TMEM245*, and *PSG4*) with evidence of distinct associations in mammary epithelial versus stromal cells that were not detected by prior GWAS^11–13^ nor TWAS^14–16,32^. These findings provide a proof of concept that cell-type-aware TWAS can reveal novel insights into the genetic and cellular etiology of human diseases.

Several recent studies have explored context-specific TWAS^35–37^. Li et al. proposed a tissue-specificity-aware TWAS framework that uses prior knowledge of trait-related tissue types for accurate detection of single-tissue and cross-tissue TWAS^35^. Feng et al. proposed to derive cross-tissue expression features using sparse canonical correlation analysis, and then combine expression-outcome associations across single- and cross-tissue features for powerful detection^36^. Thompson et al. proposed CONTENT to go one step further and model both shared and tissue-specific components of gene expression in bulk multi-tissue data for model construction^37^. This approach can also be used for modeling shared and cell-type-specific components in single-cell RNAseq data^37^. These recently developed TWAS methods model associations at the same resolution of the data, such as modeling tissue-level associations for bulk profiling data and cell-type-level associations for single-cell data, and thus do not provide higher-resolution (single cell) understanding of disease using lower-resolution (bulk tissue) data as does MiXcan.

Recent studies have also explored methods for performing cell-type-level association analyses when the tissue-level data are available for all study participants^19,38–42^. Luo et al. evaluated cell-type-specific associations between DNA methylation and traits^40^, but this method did not involve prediction models and methylation data are required for all subjects. Liu et al. built tissue-level GReX prediction models, inferred cell types from the predicted GReX, and looked for associations of the inferred cell-type proportions with disease rather than constructing a TWAS framework for identifying genes^42^. In this study, MiXcan enables cell-type-aware TWAS in large populations using existing GWAS datasets that do not have transcriptomic and cell composition data from the disease-relevant tissue. MiXcan evaluates the composite null hypothesis that there is no association between the GReX in any cell type with the disease, which tolerates decomposition uncertainty to provide robust cell-type-aware analysis using bulk tissue samples. By carefully modeling cell-type-level expression, MiXcan is more powerful than PrediXcan when disease associations are driven by a minor cell type or have opposite directions in different cell types. However, when the association of GReX with disease is similar in all cell types or driven by the major cell type, then conventional TWAS approaches using more parsimonious tissue-level GReX prediction models that assume cell-type homogeneity can be more powerful. Thus, these two TWAS approaches are complementary, and additional cell-type-aware analyses are especially valuable for diseases where cell-type heterogeneity and a minor cell of origin are hypothesized, as for breast carcinoma and many other human diseases.

To construct cell-type-level GReX prediction models, MiXcan uses a scaled xCell^18^ cell-type enrichment score in the training data as prior information. While estimates from other approaches^20,21,23,43–45^ can also be used as priors, xCell is among the most widely used. Building upon the priors, MiXcan fits mixture models for the expression levels of the epithelial cell signature genes in the training data to improve estimation of the cell-type proportion. By incorporating better estimates of the cell-type proportion, and penalizing all cell types equally, MiXcan improves the accuracy of the GReX prediction models, as well as the power and type I error of the downstream association tests. Compared with standard interaction models that include interaction effects between cell-type enrichment scores and genetic variants^19^, an important advantage of MiXcan is that the predicted values correspond to the cell-type-specific GReX, which are directly interpretable and biologically meaningful. Moreover, standard interaction models require cell composition information, which is often unavailable for the tissue of interest, whereas MiXcan prediction models can be applied directly to GWAS data without transcriptomic or cell composition data to interrogate genetic associations within the disease-relevant tissue and cell type context.

MiXcan has several limitations. First, although the MiXcan framework is generalizable, the model building procedure requires hypotheses regarding the disease-related cell types and tissue. The prediction models for this cell-type-aware TWAS of breast cancer were developed specifically for human mammary tissue with a focus on distinguishing epithelial and stromal (nonepithelial) cells, which have distinct roles in breast carcinogenesis^8–10^. Second, the current MiXcan framework decomposes a tissue into two cell-type components only. The MiXcan framework can be extended naturally to model more than two cell types. However, given limited sample sizes of the available training datasets, the model performance becomes quite variable with additional cell types due to the rapidly increasing dimension of the parameter space and complexity of the numerical optimization. As larger training sets with bulk tissue transcriptomic and genomic data become available, a careful evaluation of its analytical performance can be performed for additional less common cell types. Third, paired single-cell RNAseq and genomic datasets are presently very limited, and the validation of MiXcan epithelial cell predictions was performed for a small set of genes using epithelial cell snRNAseq data available for only three women in GTEx. Human single-cell transcriptome profiling efforts^46,47^ are currently underway, and will enable further evaluation of the performance of MiXcan in larger datasets. Future studies can also investigate integrating single-cell transcriptome profiles into MiXcan, for example to improve estimation of cell composition in bulk tissue samples^23,45^ or to provide an initial estimate of SNP weights, which could be used to tune separate penalty terms for different SNPs in adaptive elastic-net models^48^.

Human mammary tissue has variable cell composition and numerous eQTLs with distinct effects in epithelial cells and adipocytes, which are a major stromal cell type in the breast^19^. Cell-type-aware TWAS using MiXcan mammary tissue models applied to publicly available GWAS data identified three new breast cancer susceptibility genes that were associated with disease risk through their GReX in normal mammary epithelial or stromal cells. *ZNF703* (zinc finger protein 703) is an oncogene that is commonly amplified in luminal B breast tumors, and has been shown to regulate genes involved in proliferation, invasion, and an altered balance of progenitor stem cells^49–51^. To our knowledge, common germline variants in *ZNF703* have not previously been implicated in breast cancer risk. Our finding that genetic upregulation of *ZNF703* in normal mammary stromal cells (predominantly adipocytes) was associated with increased breast cancer risk in 58,648 women was confirmed by a highly significant (*p*-value=2.1 × 10^−20^) association of tissue-level GReX predicted using S-PrediXcan subcutaneous fat models in 228,951 EA women, which has not previously been reported to our knowledge. Notably, the mammary tissue-level results for *ZNF703* did not reach transcriptome-wide significance, underscoring the importance of accounting for cell-type heterogeneity to elucidate disease etiology. *TMEM245* (transmembrane protein 245) is the host gene for microRNA 32, which has been shown to promote proliferation and suppress apoptosis of breast cancer cells^52^. Relatively little is known about *PSG4* (pregnancy specific beta-1-glycoprotein 4), a member of the carcinoembryonic antigen gene family that may play a role in regulation of the innate immune system^53^. Future studies are needed to provide experimental validation of the breast cancer genes identified in this study and better understand the cellular mechanisms underlying the associations.

In conclusion, the MiXcan framework enables cell-type-aware TWAS using prediction models that allow for differences across cell types in the disease-relevant tissue. MiXcan mammary tissue models are available at https://github.com/songxiaoyu/MiXcan^54^ and can be applied to GWAS genotype data to identify genes associated with complex traits through their GReX in epithelial or stromal cells. MiXcan software is also freely available to facilitate training prediction models for other tissues and cell types, and conducting cell-type-aware TWAS. MiXcan prediction models had excellent performance in independent validation datasets, and identified new breast cancer susceptibility genes in the first cell-type-aware TWAS of breast cancer. These findings provide a proof of concept that cell-type-aware TWAS are feasible using existing bulk tissue training datasets and GWAS data, and can lead to the discovery of new disease genes and cellular mechanisms. Future research is needed to develop MiXcan models for other human tissues and cell types, extend the MiXcan framework to GWAS summary statistics, and explore alternative modeling and inference strategies^2,55,56^. These research directions will enable the broad application of cell-type-aware TWAS to improve our understanding of the genetic and cellular mechanisms underlying human diseases.

## Methods

### MiXcan framework

We first summarize PrediXcan^1^, an established tissue-level TWAS framework, and then present the MiXcan cell-type-aware TWAS framework. Let *y*_*i*_ denote the measured expression level of a gene in the bulk tissue sample *i* ∈ (1, . . . , *N*), **x**_*i*_ denote the genetic variants (e.g. SNPs) used in model training, and **z**_*i*_ denote the non-genetic covariates (e.g. age).

#### PrediXcan tissue-level TWAS framework

PrediXcan uses a linear additive model to characterize the gene expression level:

1 $${y}_{i}=a+{{{{{{{{\bf{x}}}}}}}}}_{i}^{T}{{{{{{{\bf{b}}}}}}}}+{{{{{{{{\bf{z}}}}}}}}}_{i}^{T}{{{{{{{\bf{c}}}}}}}}+{e}_{i},$$

where *e*_*i*_ ~ *N*(0, *σ*^2^) is the model error, ${{{{{{{{\bf{x}}}}}}}}}_{i}^{T}{{{{{{{\bf{b}}}}}}}}$ is the genetically determined component of gene expression, and ${{{{{{{{\bf{z}}}}}}}}}_{i}^{T}{{{{{{{\bf{c}}}}}}}}$ is the non-genetically determined component. This model can be estimated with the elastic-net method^57^, which maximizes the following penalized log-likelihood function:

2 $$(\widehat{a},\, \widehat{{{{{{{{\bf{b}}}}}}}}},\, \widehat{{{{{{{{\bf{c}}}}}}}}},\, \widehat{\sigma })=	 \arg \max \mathop{\sum }\limits_{i=1}^{n}\left(-\frac{{({y}_{i}-a-{{{{{{{{{\bf{x}}}}}}_{i}^{T}}}}}{{{{{{{\bf{b}}}}}}}}-{{{{{{{{{\bf{z}}}}}}_{i}^{T}}}}}{{{{{{{\bf{c}}}}}}}})}^{2}}{2{\sigma }^{2}}-\frac{1}{2}\log ({\sigma }^{2})\right)\\ 	-\lambda (P({{{{{{{\bf{b}}}}}}}})+P({{{{{{{\bf{c}}}}}}}})),$$

where $P(\cdot )=\alpha||\cdot|{|}_{{l}_{2}}^{2}+(1-\alpha )||\cdot|{|}_{{l}_{1}}$ is the elastic-net penalty function with the mixing parameter *α* ∈ (0, 1). In PrediXcan, *α* is set at 0.5 and *λ* is selected via 10-fold cross validation.(CV)^58^ The estimated SNP weights $\widehat{{{{{{{{\bf{b}}}}}}}}}$ can be used to predict the GReX by ${\widehat{y}}_{j}={\tilde{x}}_{j}^{T}\widehat{{{{{{{{\bf{b}}}}}}}}}$ where ${\tilde{x}}_{j}$ denotes the SNP genotypes of the GWAS subject *j* ∈ (1, . . . , *M*). Then, the association of ${\widehat{y}}_{j}$ with the phenotype (e.g. disease status) *d*_*j*_ can be evaluated using a generalized linear model $g({d}_{j})={\eta }_{0}+{\widehat{y}}_{j}{\eta }_{1}$, where *g*(. ) is a link function. The null and alternative hypotheses, *H*_0_: *η*_1_ = 0 vs. *H*_*A*_: *η*_1_ ≠ 0, test whether the GReX at the tissue level is associated with the phenotype.

#### MiXcan cell-type-level GReX prediction model

MiXcan extends upon PrediXcan to enable cell-type-aware TWAS. In this section, we build the prediction models for the cell-type-level GReX. In the next section, we develop strategies for applying these prediction models in disease-association studies.

Human bulk tissue samples from solid tissue (e.g. mammary tissue) comprise a mixture of cells of different types. Let *π*_*i*_ and 1 − *π*_*i*_ be the proportions of the cell type of interest (e.g. epithelial cells) and all other cell types (e.g. stromal cells) in the *i*^*th*^ tissue sample, respectively. We assume the observed bulk tissue expression level of a given gene *y*_*i*_ is a linear combination of expression levels in the cell types. Introducing two latent variables *u*_*i*_ and *v*_*i*_ to denote the unobserved average gene expression levels in the epithelial and stromal cells, respectively, we have:

3 $${y}_{i}={\pi }_{i}{u}_{i}+(1-{\pi }_{i}){v}_{i}.$$

We model both *u*_*i*_ and *v*_*i*_ using the linear additive models:

4 $$\begin{array}{rcl}{u}_{i}={a}_{u}+{{{{{{{{\bf{x}}}}}}}}}_{i}^{T}{{{{{{{{\bf{b}}}}}}}}}_{u}+{{{{{{{{\bf{z}}}}}}}}}_{i}^{T}{{{{{{{\bf{c}}}}}}}}+{e}_{ui},\quad {{{{{{{\rm{and}}}}}}}} \quad {v}_{i}={a}_{v}+{{{{{{{{\bf{x}}}}}}}}}_{i}^{T}{{{{{{{{\bf{b}}}}}}}}}_{v}+{{{{{{{{\bf{z}}}}}}}}}_{i}^{T}{{{{{{{\bf{c}}}}}}}}+{e}_{vi},\end{array}$$

where ${e}_{ui} \sim N(0,\, {\sigma }_{u}^{2})$ and ${e}_{vi},\, \sim N(0,{\sigma }_{v}^{2})$ are the model errors. In Eq. (4), the intercepts *a*_*u*_ and *a*_*v*_ and genetic parameters **b**_*u*_ and **b**_*v*_ differ between cell types, allowing for different mean expression levels and cell-type-specific effects of genetic variants on gene expression. A shared parameter **c** is used for the non-genetic component **z**_*i*_ to simplify the model as the non-genetic variables are not necessarily used in downstream analyses.

The proportion of epithelial cells ***π*** = {*π*_1_, . . . , *π*_*N*_} is a feature of the tissue samples, which can be jointly estimated using multiple genes, whereas ${{{{{{{\boldsymbol{\Theta }}}}}}}}=({a}_{u},\, {a}_{v},\, {{{{{{{{\bf{b}}}}}}}}}_{u},\, {{{{{{{{\bf{b}}}}}}}}}_{v},\, {{{{{{{\bf{c}}}}}}}},\, {\sigma }_{u}^{2},\, {\sigma }_{v}^{2})$ are features of each gene for investigation. Therefore, we present a step-wise procedure to first estimate ***π*** using multiple epithelial signature genes, and then estimate the cell-type-level effects **Θ** for each gene.

**Estimation of** ***π***. The notation in the sections above focuses on individual genes, and here we introduce additional notation to describe the joint modeling of multiple genes. Let ${W}_{N\times G}=\{{w}_{i}^{g}\}$ be the observed expression of *G* epithelial signature genes in *N* tissue samples; and ${S}_{N\times G}=\{{s}_{i}^{g}\}$ and ${T}_{N\times G}=\{{t}_{i}^{g}\}$ be the unobserved gene expression levels in epithelial and stromal cells, respectively. Similarly as in Eq. (3), we have:

5 $${w}_{i}^{g}={\pi }_{i}{s}_{i}^{g}+(1-{\pi }_{i}){t}_{i}^{g},\quad g=1,\, ...,\, G,\quad i=1,\, ...,\, N.$$

Leveraging primarily the mean differences of signature genes in epithelial and stromal cells for estimating ***π***, we model the marginal distributions of individual genes and omit the complex gene-gene correlations for computationally efficient estimation, as supported by our previous work^59^. Specifically, we assume:

$${s}_{i}^{g} \sim N({\mu }_{Si}^{g},\, {\sigma }_{Si}^{g}),\quad {{{{{{{\rm{and}}}}}}}} \, {t}_{i}^{g} \sim N({\mu }_{Ti}^{g},\, {\sigma }_{Ti}^{g}).$$

Across all *G* genes, the parameters include ${{{{{{{\boldsymbol{\Gamma }}}}}}}}={\{\{{{{{{{{{\boldsymbol{\Gamma }}}}}}}}}^{g}\}\}}_{g=1}^{G}$, where ${{{{{{{{\boldsymbol{\Gamma }}}}}}}}}^{g}=({{{{{{{{\boldsymbol{\mu }}}}}}}}}_{S}^{g},\, {{{{{{{{\boldsymbol{\mu }}}}}}}}}_{T}^{g},\, {\sigma }_{S}^{g},\, {\sigma }_{T}^{g})$.

In parallel, we also take advantage of a prior cell-type proportion estimate *h*_*i*_ based on existing tools (i.e. rescaled xCell^18^ enrichment scores). We link the prior estimates *h*_*i*_ to the true *π*_*i*_ using a *Beta* distribution such that *h*_*i*_ ~ *Beta*(*π*_*i*_*δ*, (1 − *π*_*i*_)*δ*) for some positive parameter *δ*. We have *E*(*h*_*i*_) = *π*_*i*_ and *var*(*h*_*i*_) = *π*_*i*_(1 − *π*_*i*_)/(*δ* + 1) such that *h*_*i*_ is an unbiased estimator of the true *π*_*i*_ with variation.

We then join these two models for parameter estimation and solve the following maximization problem:

6 $$(\hat{{{{{{{{\boldsymbol{\pi }}}}}}}}},\hat{{{{{{{{\boldsymbol{\Gamma }}}}}}}}},\hat{\delta })=\arg \mathop{\max }\limits_{{{{{{{{\boldsymbol{\Gamma }}}}}}}},{{{{{{{\boldsymbol{\pi }}}}}}}},\delta }\mathop{\sum }\limits_{i=1}^{N}\left[\mathop{\sum }\limits_{g=1}^{G}l({{{{{{{\boldsymbol{\Gamma }}}}}}}},\, {\pi }_{i}|{w}_{i}^{g})+l({\pi }_{i},\, \delta|{h}_{i})\right],$$

where $\mathop{\sum }\nolimits_{g=1}^{G}l({{{{{{{\boldsymbol{\Gamma }}}}}}}},\, {\pi }_{i}|{w}_{i}^{g})$ and *l*(*π*_*i*_, *δ*∣*h*_*i*_) are the log-likelihood of the observed gene expression profile and cell proportion estimate of the *i*^*th*^ sample, respectively. This optimization problem is solved using an Expectation-Maximization (EM) algorithm similar to that in Petralia et al.^59^

To enhance the robustness of the estimation, we implement a bagging strategy to estimate the parameters with randomly selected bootstrap samples, and aggregate multiple estimates by calculating a tail truncated mean. This bagging strategy further stabilizes the estimates, and may also be used to investigate the consistency of ***π*** estimation.

**Estimation ofb**_*u*_**andb**_*v*_. Given $\widehat{{{{{{{{\boldsymbol{\pi }}}}}}}}}$, we next estimate **b**_*u*_ and **b**_*v*_ in Eq. (4). Since *u*_*i*_ and *v*_*i*_ are unobserved, we integrate Eqs. (3) and (4) to have:

7 $${y}_{i}={\widehat{\pi }}_{i}({a}_{u}+{{{{{{{{\bf{x}}}}}}}}}_{i}^{T}{{{{{{{{\bf{b}}}}}}}}}_{u}+{{{{{{{{\bf{z}}}}}}}}}_{i}^{T}{{{{{{{\bf{c}}}}}}}}+{e}_{ui})+(1-{\widehat{\pi }}_{i})({a}_{v}+{{{{{{{{\bf{x}}}}}}}}}_{i}^{T}{{{{{{{{\bf{b}}}}}}}}}_{v}++ {{{{{{{{\bf{z}}}}}}}}}_{i}^{T}{{{{{{{\bf{c}}}}}}}}+{e}_{vi}).$$

This equation can be rearranged as:

8 $${y}_{i}={a}_{v}+{\widehat{\pi }}_{i}({a}_{u}-{a}_{v})+{{{{{{{{\bf{x}}}}}}}}}_{i}^{T}{{{{{{{{\bf{b}}}}}}}}}_{v}+{\widehat{\pi }}_{i}{{{{{{{{\bf{x}}}}}}}}}_{i}^{T}({{{{{{{{\bf{b}}}}}}}}}_{u}-{{{{{{{{\bf{b}}}}}}}}}_{v})+{{{{{{{{\bf{z}}}}}}}}}_{i}^{T}{{{{{{{\bf{c}}}}}}}}+{\epsilon }_{i},$$

where ${\epsilon }_{i}={\widehat{\pi }}_{i}{e}_{ui}+(1-{\widehat{\pi }}_{i}){e}_{vi}$. A simple strategy for estimating **b**_*u*_ and **b**_*v*_ is to apply elastic-net regression to Eq. (8). Specifically, the elastic-net regularization is put on (**b**_*v*_, **b**_*u*_ − **b**_*v*_, **c**)—the dependence of expression levels on genetic variants and covariates—but not on (*a*_*v*_, *a*_*u*_ − *a*_*v*_) —the mean expression levels in the two cell types. We refer to this strategy as MiXcan_0_.

One issue with MiXcan_0_ is that the two cell components in the mixture model are not treated in a symmetric manner. In other words, the penalization on **b**_*u*_ and **b**_*v*_ differs: **b**_*v*_ is shrunk towards zero, while **b**_*u*_ is shrunk towards **b**_*v*_. This asymmetric penalization results in different models if the order of the two components is switched. To address this issue, we introduce ${\widehat{c}}_{i}={\widehat{\pi }}_{i}-0.5$ and rewrite Eq. (8) as:

9 $${y}_{i}=\frac{{a}_{u}+{a}_{v}}{2}+{\widehat{c}}_{i}({a}_{u}-{a}_{v})+{{{{{{{{\bf{x}}}}}}}}}_{i}^{T}\frac{{{{{{{{{\bf{b}}}}}}}}}_{u}+{{{{{{{{\bf{b}}}}}}}}}_{v}}{2}+{\widehat{c}}_{i}{{{{{{{{\bf{x}}}}}}}}}_{i}^{T}({{{{{{{{\bf{b}}}}}}}}}_{u}-{{{{{{{{\bf{b}}}}}}}}}_{v})+{{{{{{{{\bf{z}}}}}}}}}_{i}^{T}{{{{{{{\bf{c}}}}}}}}+{\epsilon }_{i}.$$

When fitting elastic-net regression to Eq. (9), we include penalties on $(\frac{{{{{{{{{\bf{b}}}}}}}}}_{u}+{{{{{{{{\bf{b}}}}}}}}}_{v}}{2},\, ({{{{{{{{\bf{b}}}}}}}}}_{u}-{{{{{{{{\bf{b}}}}}}}}}_{v}),{{{{{{{\bf{c}}}}}}}})$, which impose the same degree of regularization on **b**_*u*_ and **b**_*v*_: the penalty on $\frac{{{{{{{{{\bf{b}}}}}}}}}_{u}+{{{{{{{{\bf{b}}}}}}}}}_{v}}{2}$ regularizes the overall sparsity of the genetic effects, while the penalty on **b**_*u*_ − **b**_*v*_ encourages similarities between the two components. We refer to this strategy as MiXcan.

Note, when fitting elastic-net regressions in MiXcan_0_ and MiXcan, we do not consider the varying variances of *ϵ*_*i*_ as ${\hat{\pi }}_{i}$ takes different values. This is because the residual variance structure has limited impact on the coefficient estimates, especially for regularized regression. In a trade-off between extensive computational costs (allowing varying residual variances) and minimal sacrifice of estimation accuracy (assuming constant variance), we chose the latter and take advantage of the fast implementation of elastic-net regression in the *glmnet* package.

**Model Aggregation**. In the prediction models, the term ${\widehat{{{{{{{{\bf{b}}}}}}}}}}_{u}-{\widehat{{{{{{{{\bf{b}}}}}}}}}}_{v}$ is of particular importance: a non-zero value suggests that the dependence structure between genetic variants and expression levels is cell-type-specific. Therefore, it is critical to know the selection robustness of ${\widehat{{{{{{{{\bf{b}}}}}}}}}}_{u}-{\widehat{{{{{{{{\bf{b}}}}}}}}}}_{v}$. We employ a procedure similar to stability selection^60^ for its evaluation. Specifically, for models that select non-zero ${\widehat{{{{{{{{\bf{b}}}}}}}}}}_{u}-{\widehat{{{{{{{{\bf{b}}}}}}}}}}_{v}$, we generate *B* bootstrap samples (e.g. *B* = 200), perform ordinary least square analysis on the pre-selected variables, and record ${\widehat{{{{{{{{\rm{diff}}}}}}}}}}_{{{{{{{{\bf{b}}}}}}}}}^{(b)}={\widehat{{{{{{{{\bf{b}}}}}}}}}}_{u}^{(b)}-{\widehat{{{{{{{{\bf{b}}}}}}}}}}_{v}^{(b)}$ for *b* = 1, . . . , *B*. Only when the 95% confidence interval (CI) for ${\widehat{{{{{{{{\rm{diff}}}}}}}}}}_{{{{{{{{\bf{b}}}}}}}}}$ excludes 0 do we employ cell-type-specific prediction models (inferred using the complete data set). Otherwise, nonspecific models that have the same prediction weights for the two cell types will be used, as in Eq. (2) of PrediXcan.

#### Association analysis with cell-type-level prediction models

The model building procedure in MiXcan selects cell-type-specific prediction models for some genes and nonspecific models for other genes. Cell-type-specific prediction models estimate different SNP weights in the two cell types (${\widehat{{{{{{{{\bf{b}}}}}}}}}}_{u} \, \ne \, {\widehat{{{{{{{{\bf{b}}}}}}}}}}_{v}$) resulting in different predicted GReX from the same genotype data ${\tilde{x}}_{j}$ (*j* ∈ (1, ..., *M*)), such that ${\tilde{y}}_{uj}={\tilde{x}}_{j}^{T}{\widehat{{{{{{{{\bf{b}}}}}}}}}}_{u}$ and ${\tilde{y}}_{vj}={\tilde{x}}_{j}^{T}{\widehat{{{{{{{{\bf{b}}}}}}}}}}_{v}$. These cell-type-specific GReX levels cannot be combined into tissue levels in GWAS datasets that lack cell-type proportion estimates, requiring a novel statistical framework for association analysis. One natural idea is to infer cell-type-specific associations by directly associating the phenotype *d*_*j*_ with ${\tilde{y}}_{uj}$ and ${\tilde{y}}_{vj}$, either separately, such that $g({d}_{j})={\eta }_{0}+{\eta }_{u}{\tilde{y}}_{uj}$ and $g({d}_{j})=\eta {{\prime} }_{0}+{\eta }_{v}{\tilde{y}}_{vj}$, or jointly, such that $g({d}_{j})={\eta }_{0}+{\eta }_{u}{\tilde{y}}_{uj}+{\eta }_{v}{\tilde{y}}_{vj}$. As ${\tilde{y}}_{uj}$ and ${\tilde{y}}_{vj}$ are predicted from the same genotype data with prediction weights jointly learned using the bulk tissue data in MiXcan, they may capture leaked information from each other. As a result, if an association exists in one cell type, analysis in the other cell type may also capture this association, resulting in an inflated type I error for inferring associations in each cell type. To avoid this inflation, we propose a composite hypothesis test to test whether association exists in any cell types in the tissue:

- *H*_0_: There is no association in any cell type in the tissue vs.
- *H*_*A*_: There is an association in at least one cell type in the tissue.

This composite null is robust against information leakage, as the leaked values under the null are not associated with the phenotype. To perform the test, we first associate *d*_*j*_ with ${\tilde{y}}_{uj}$ and ${\tilde{y}}_{vj}$, separately if the $({\tilde{y}}_{uj},{\tilde{y}}_{vj})$ are highly correlated (e.g. *r* = ±1), or jointly otherwise. Then, we propose to aggregate the resulting *p* values *p*_*u*_ and *p*_*v*_ for ${\tilde{y}}_{uj}$ and ${\tilde{y}}_{vj}$ using Cauchy combination^27^. The Cauchy combination provides valid test for correlated *p*-values, and in this setting the test statistic can be written as:

10 $${T}_{{Cauchy}}=\tan \{{{{{{{{\rm{pi}}}}}}}}(0.5-{p}_{u})\}+\tan \{{{{{{{{\rm{pi}}}}}}}}(0.5-{p}_{v})\},$$

where pi is the mathematical constant approximately equal to 3.14159. The combined *p*-value for the tissue is approximated by:

11 $${p}_{{tissue}} \, \approx \, 1/2-\{\arctan ({T}_{Cauchy})\}{{{{{{{\rm{pi}}}}}}}}.$$

The *p*_*tissue*_ tests whether association exists in any cell type in the tissue. Unlike PrediXcan that tests associations averaged across all cell types, the *p*_*tissue*_ in MiXcan accumulates signals from different cell types. Note that *p*_*u*_ and *p*_*v*_ are building blocks of *T*_*Cauchy*_ and the resulting *p*_*tissue*_ is between *p*_*u*_ and *p*_*v*_. After the tissue-level hypothesis test, *p*_*u*_ and *p*_*v*_ can provide information on the cell type(s) driving the association. For example, *p*_*u*_ << *p*_*v*_ indicates that a significant *p*_*tissue*_ is primarily driven by epithelial cells.

Some genes have nonspecific models with the same estimated SNP weights and predicted GReX in the two cell types (${\widehat{{{{{{{{\bf{b}}}}}}}}}}_{u}={\widehat{{{{{{{{\bf{b}}}}}}}}}}_{v}$). While association analyses can follow the same strategy described above for cell-type-specific models, it is equivalent to performing a single tissue-level association analysis as in PrediXcan. Finally, in transcriptome-wide studies, *p*_*tissue*_ from genes with cell-type-specific prediction models and *p*-values from genes with nonspecific models can be jointly used to adjust for multiple testing, and infer transcriptome-wide significant discoveries.

### Build MiXcan prediction models using GTEx mammary tissue data

MiXcan gene expression prediction models were developed using the GTEx v8 genotype and gene expression data for mammary tissue samples from 125 European ancestry women (dbGaP accession number phs000424.v8.p2 <https://www.ncbi.nlm.nih.gov/projects/gap/cgi-bin/study.cgi?study_id=phs000424.v8.p2>). PredictDB (http://predictdb.org) provides tissue-level expression prediction models trained on 337 men and women of European ancestry with mammary tissue data available in GTEx v8. PredictDB included mammary tissue elastic-net models for a total of 6461 genes that were well predicted by genetic variants (176,983 SNPs). An additional 1,715 SNPs were included in PredictDB mammary tissue MASHR models^61^. For the purpose of comparison to PrediXcan as a proof of concept, we developed cell-type-level prediction models for these 6461 genes using all 178,698 SNPs in the PredictDB mammary tissue database. MiXcan cell-type-level prediction models were developed for mammary epithelial cells, the cell of origin for breast carcinoma, and stromal (non-epithelial) cells.

***π* estimation**. The epithelial cell proportion ***π***_*i*_ was estimated using 126 epithelial cell signature genes^18^ available in the training dataset of 125 GTEx female mammary tissue samples. We first computed xCell gene set enrichment scores for epithelial cells and 63 other cell types using the curated set of cell signature genes for each cell type^18^ and the bulk tissue transcriptomic data for each sample. We then re-scaled the xCell epithelial cell enrichment score to range from 0 to 1 for use as a prior estimate of the cell-type proportion in MiXcan. The ***π***_*i*_ estimation was performed using 100 bootstrap samples (80% random draw with replacement). The final estimate was computed by excluding the most extreme 5% of bootstrap estimates in each of the two tails and averaging the remaining estimates.

**Prediction model**. Using $\widehat{{{{{{{{\boldsymbol{\pi }}}}}}}}}$, we modeled the cell-type-level expression levels for each of the 6461 genes using MiXcan with tuning parameter *λ* selected by tenfold CV. We adjusted for covariates that were used in GTEx eQTL analyses including age, platform, PCR, genomic principal components (PC) 1-5, and PEER factors 1–15^62^. For genes with ${\widehat{{{{{{{{\bf{b}}}}}}}}}}_{u} \, \ne \, {\widehat{{{{{{{{\bf{b}}}}}}}}}}_{v}$, we performed ordinary least squares regression on the pre-selected variables for 200 bootstrap samples, and calculated the 95% bootstrap CI of ${\widehat{{{{{{{{\bf{b}}}}}}}}}}_{u}-{\widehat{{{{{{{{\bf{b}}}}}}}}}}_{v}$. If the 95% CI excluded 0, we used cell-type-specific models with parameters estimated using the full data; otherwise, we used nonspecific models that were the same as PrediXcan. The average computation time for training models for 1000 genes using 125 samples on a single CPU core was 11 min (standard deviation, 2.8 minutes).

### Evaluate MiXcan prediction accuracy in independent TCGA data

We evaluated the prediction performance of MiXcan in an independent dataset of 103 European ancestry female breast cancer patients with adjacent normal tissue samples from TCGA^63,64^. To minimize the study effect, we re-processed the TCGA gene expression data using methods analogous to those used to process the GTEx expression data (https://gtexportal.org/home/documentationPage). Briefly, we required genes to have Transcripts Per Kilobase Million (TPM) >0.1 in at least 20% of samples, and at least six reads in at least 20% of samples, resulting in a set of 25,702 out of 25,849 total genes that met these quality control (QC) requirements. Expression data were then normalized using the trimmed mean of M values method (TMM)^65^ as implemented in the R package edgeR v3.16.5^66^, and the results were quantile-normalized to a standard normal distribution with mean=0 and variance = 1. Comparison of these normalized gene expression levels showed no systematic differences between the GTEx and TCGA data (Supplementary Fig. 7). To process the genotype data, we removed all indels, monomorphisms, and ambiguous pairs (e.g. A/T, C/G). SNPs with >5% missing genotypes or Hardy-Weinberg equilibrium (HWE) test *p* value < 1e–05 were also removed. The remaining SNPs were aligned to build 37 coordinates, and imputation was performed on the TOPMed imputation server^67^. A total of 97% (54,663 out of 56,531) and 97% (52,031 out 53,876) SNPs used in MiXcan and PrediXcan prediction models, respectively, were available for analysis.

We estimated the epithelial cell proportion in the TCGA samples as described above, and used this estimate to combine the predicted cell-type-level GReX from epithelial and stromal components into the tissue level. To evaluate predication accuracy, we computed the Pearson correlation between the predicted and observed tissue-level gene expression, and compared the results with the predicted tissue-level expression using PrediXcan. The observed bulk tissue expression levels showed significantly higher correlation with the tissue-level GReX predicted by MiXcan compared with PrediXcan. To investigate the sources of the improved performance, we compared five approaches for predicting tissue-level GReX:

- Existing PredictDB elastic-net models (PredictDB)
- PrediXcan trained on the same dataset as MiXcan (PrediXcan)
- Prediction model including interactions between SNPs and the xCell enrichment score (xCell interaction)
- Prediction model including interactions between SNPs and the MiXcan cell-type proportion (MiXcan_0_)
- Cell-type-level prediction models with symmetric penalization (MiXcan).

These comparisons evaluated incorporating cell-type composition, use of the MiXcan cell-type proportion estimate, and symmetric penalization of the two cell types in prediction models, as well as use of a larger training set including both men and women in PredictDB. It is worth noting that “MiXcan_0_" and “xCell interaction" models are not applicable to GWAS datasets that lack cell-type composition information for the tissue of interest, and are included here only for the purpose of understanding the sources of improved prediction performance for MiXcan.

### Evaluate MiXcan epithelial cell prediction accuracy in snRNAseq data

We evaluated the performance of MiXcan epithelial cell prediction models using snRNAseq data for normal mammary epithelial cells and paired genomic data available for three women of European, Asian and African ancestry from GTEx v9^29^. Details of the snRNAseq data generation and processing were provided in^29^. The preprocessed log count expression profiles for a total of 5990, 2324 and 1456 nuclei, including 2292 (38%), 2180 (94%) and 1327 (91%) epithelial cell nuclei, from the European, Asian and African ancestry woman, respectively, were downloaded from https://gtexportal.org/home/datasets. Mammary epithelial cell snRNAseq data were available in all three women for 4751 genes with MiXcan prediction models. To enable comparisons between women, the snRNAseq levels were averaged for each gene and quantile normalized to a standard normal distribution within each woman to reduce the impact of noise, skewness and outliers.

To evaluate prediction accuracy, MiXcan epithelial cell prediction models were applied to the genotype data for each woman, and the between-woman difference in the predicted GReX for each gene was computed to identify the two sets of 100 genes predicted to have the largest positive or negative differences in mammary epithelial cell expression in each pair of women. The Wilcoxon signed-rank test was used to test whether the observed snRNAseq differences for genes predicted to have the largest GReX differences between women were significantly different from zero, as expected.

### Simulation studies

To evaluate the type I error and power of MiXcan association tests, we performed extensive simulation studies under a broad range of realistic models for the associations of genetic variants with gene expression (SNP-Exp) and gene expression with disease (Exp-Disease). Mimicking real data, in each simulation, we generated a training dataset for building the GReX prediction models, and a GWAS dataset for testing the associations of GReX with disease. Without loss of generality, non-genetic covariates were excluded from simulations to allow direct evaluation of the predicted GReX.

For the training dataset, we simulated 300 bulk tissue samples with observed SNP genotypes and tissue-level gene expression. We assumed each tissue *i* ∈ (1, . . . , 300) was a mixture of two cell types and that the minor cell type (cell type 1) comprised an average of 40% of the tissue, with proportion *π*_*i*_ ~ *Beta*(*α* = 2, *β* = 3). We further simulated the genotypes of 50 neighboring SNPs **x**_*i*_ = {*x*_1*i*_, . . . , *x*_50*i*_} using the genome simulator R package *sim1000G*, with its default reference genome region (chromosome 4) and minor allele frequency (MAF) range 0.05–0.50. For the expression levels in the two cell types *u*_*i*_ and *v*_*i*_, we considered a linear additive model such that *u*_*i*_ = *b*_0_ + **b**_1_**x**_*i*_ + *e*_*ui*_ and *v*_*i*_ = **b**_2_**x**_*i*_ + *e*_*vi*_ where *e*_*ui*_, *e*_*vi*_ ~ *N*(0, 1). The parameter *b*_0_ determined the mean expression difference in the two cell types under **x**_*i*_ = 0, and **b**_1_ and **b**_2_ determined the association patterns between the SNPs *X* and gene expression level *Y* in the minor and major cell types, respectively. Then, the tissue-level gene expression was a weighted average of the expression levels in the two cell types: *y*_*i*_ = *π*_*i*_*u*_*i*_ + (1 − *π*_*i*_)*v*_*i*_.

We considered two SNP-Exp settings: *Homogeneous SNP-Exp Association* (**b**_1_ = **b**_2_) and *Heterogeneous SNP-Exp Association* (**b**_1_ ≠ **b**_2_). Under the *Homogeneous SNP-Exp* setting, we randomly selected one genetic variant *p* to be associated with expression levels in the two cell types and let *b*_1*p*_ = *b*_2*p*_ = 1 or -1 with equal chance, corresponding to a median heritability of 0.27 and interquartile range (IQR) of 0.10. We varied *b*_0_ from -2 to 2 to evaluate the impact of the intercept (mean expression difference in two cell types under **x**_*i*_ = 0) on TWAS. Under the Heterogeneous SNP-Exp setting, we randomly selected two SNPs *p*_1_ ≠ *p*_2_ ∈ (1, . . . , 50) with SNPs *p*_1_ and *p*_2_ associated with expression levels in the minor and major cell types, respectively, corresponding to the same heritability in both cell types (median 0.27; IQR 0.10). Similar to the Homogeneous SNP-Exp setting, we first evaluated the impact of the intercept by varying *b*_0_ from -2 to 2 while fixing ${b}_{1{p}_{1}}$ at 1 or -1 and ${b}_{2{p}_{2}}$ at 1 or -1 with equal chance. Second, we varied the magnitude of *b*_1*p*_ from 0 to 2 (median heritability 0 to 0.59) with equal chance of a positive or negative sign, while fixing *b*_0_ at 1 and *b*_2*p*_ = ± 1 to understand the impact of the SNP-Exp association strength in the minor cell type. Third, we varied the magnitude of *b*_2*p*_ from 0 to 2 (allowing a random sign with equal chance), while fixing *b*_0_ at 1 and *b*_1*p*_ = ± 1 to understand the impact of the SNP-Exp association strength in the major cell type. Finally, to evaluate the impact of the training data sample size, we assessed sample sizes ranging from 100 to 300, while fixing *b*_0_ at 1 and the magnitude of non-zero components of **b**_1_, **b**_2_ at 1.

For the GWAS dataset, we simulated a case-control study of 3000 participants with observed genotypes and disease status. We assumed the unobserved cell-type composition and cell-type-level gene expression in this dataset followed the same distributions as in the training dataset. Disease risk was simulated using a logistic model, such that *logitP*(*d*_*j*_ = 1) = *η*_0_ + *η*_1_*u*_*j*_ + *η*_2_*v*_*j*_ for *j* ∈ (1, . . . , 3000). The intercept *η*_0_ was set to reflect a 1:1 ratio of cases and controls. We considered five different settings for *η*_1_, *η*_2_ to capture the dynamic relationship between gene expression levels in the two cell types and disease risk:

1. No Exp-Disease Association: *η*_1_ = *η*_2_ = 0, i.e. disease is not associated with the gene expression in either cell type.
2. Homogeneous Exp-Disease Association: *η*_1_ = *η*_2_ = 0.2, i.e. disease is associated with the gene expression in both cell types in the same way.
3. Exp-Disease Association in Major Cell: *η*_1_ = 0 and *η*_2_ = 0.2, i.e. disease is associated with the gene expression in the major cell type (cell type 2).
4. Exp-Disease Association in Minor Cell: *η*_1_ = 0.2 and *η*_2_ = 0, i.e. disease is associated with the gene expression in the minor cell type (cell type 1).
5. Exp-Disease Association in Opposite Directions: *η*_1_ = − 0.2 and *η*_2_ = 0.2, i.e. disease is associated with the gene expression in the two cell types in opposite directions.

We compared the prediction accuracy, type I error, and power of MiXcan with PrediXcan, which ignores cell-type heterogeneity in 500 Monte Carlo simulations.

**MiXcan performance under misspecified models**. MiXcan decomposes tissues into two cell-type components. To evaluate the robustness of MiXcan to misspecification of the cell-type proportion, where a noisy estimate of $\hat{{{{{{{{\boldsymbol{\pi }}}}}}}}}$ is used instead of the true ***π***, we simulated: (a) $\hat{{{{{{{{\boldsymbol{\pi }}}}}}}}}=0.8{{{{{{{\boldsymbol{\pi }}}}}}}}$ to shift the mean from 0.4 to 0.32 and scale from (0–1) to (0–0.8); (b) $\hat{{{{{{{{\boldsymbol{\pi }}}}}}}}}=0.7{{{{{{{\boldsymbol{\pi }}}}}}}}+0.2$ to shift the mean from 0.4 to 0.48 and scale from (0-1) to (0.2-1); (c) ${\hat{\pi }}_{i} \sim Beta(50{\pi }_{i},50(1-{\pi }_{i}))$ to reduce the correlation with the true value $cor({\hat{\pi }}_{i},{\pi }_{i})$ to 0.9; and (d) ${\hat{\pi }}_{i} \sim Beta(5.5{\pi }_{i},5.5(1-{\pi }_{i}))$ to further reduce $cor({\hat{\pi }}_{i},{\pi }_{i})$ to 0.6. We compared the performance of MiXcan using the misspecified $\hat{{{{{{{{\boldsymbol{\pi }}}}}}}}}$ with MiXcan using the true ***π*** and PrediXcan.

We also evaluated the robustness of MiXcan to the presence of a latent third cell type. We simulated a tissue with three cell types that have different SNP-Exp or Exp-Disease associations, and evaluated the performance of MiXcan by decomposing the tissue into cell type 1 vs. a mixture of cell types 2 and 3. Specifically, we simulated 300 bulk tissue samples comprised of three cell types with proportions *π*_1*i*_ = 40%, *π*_2*i*_ = 50% and *π*_3*i*_ = 10%. As in the simulations above, gene expression levels in three cell types were linearly dependent on 50 neighboring SNPs as determined by **b**_1_, **b**_2_ and **b**_3_, and logit transformed disease risk was linearly dependent on the expression levels in the three cell types as determined by *η*_1_, *η*_2_ and *η*_3_. To evaluate the impact of a latent third cell type contributing to the SNP-Exp association, we compared the performance of MiXcan and PrediXcan for **b**_2_ = **b**_3_ vs. **b**_2_ ≠ **b**_3_ assuming *η*_2_ = *η*_3_ under the Heterogeneous SNP-Exp Association (**b**_1_ ≠ **b**_2_) setting. In detail, we randomly selected three different SNPs *p*_1_, *p*_2_, *p*_3_ ∈ (1, . . . , 50) to be associated with expression levels in the three cell types, respectively, and fixed the magnitude of these non-zero **b** (${b}_{1{p}_{1}},\, {b}_{2{p}_{2}},\, {b}_{2{p}_{3}}$) at 1, with equal chance of a positive or negative sign. We evaluated type I error and power in simulated GWAS datasets (N = 3000) under five Exp-Disease patterns as described for *η*_1_, *η*_2_ above. As we observed that type I error was well controlled under various SNP-Exp associations in a latent third cell type, we next evaluated the impact of a latent third cell type contributing to the Exp-Disease association on the study power of MiXcan. In this simulation, we fixed **b**_3_ = **b**_2_ but simulated *η*_3_ ≠ *η*_2_ with *η*_3_ values ranging from -0.2 to 0.2.

### Apply MiXcan to perform cell-type-aware TWAS of breast cancer

#### Cell-type-aware TWAS of breast cancer

We performed cell-type-aware TWAS of breast cancer risk using GWAS data from the Discovery, Biology, and Risk of Inherited Variants in Breast Cancer (DRIVE) study. Genotype data for 60,014 women (32,438 cases and 27,576 controls) assayed using the Oncoarray^30^, which includes >500,000 variants and provides excellent coverage of most common variants, were downloaded from dbGaP (phs001265.v1.p1 <https://www.ncbi.nlm.nih.gov/projects/gap/cgi-bin/study.cgi?study_id=phs001265.v1.p1>). After imputation (as described above for TCGA data), 95% (53,528 out of 56,531) and 95% (51,049 out of 53,876) SNPs used in MiXcan and PrediXcan prediction models, respectively, were available for analysis.

Principle component analysis (PCA) was performed using 20,629 SNPs, after excluding SNPs with a missing rate above 0.01% and selecting SNPs in approximate linkage equilibrium using PLINK v1.90 (indep-pairwise option with window size=50kb, step size=5, *r*^2^ threshold=0.05)^68^. EIGENSOFT v6.1.4 was used to compute PCs with the fast mode option enabled, which implements the FastPCA approximation^69^. The first PC separated individuals of African (e.g. from Nigeria, Uganda and Cameroon) vs. European (e.g. from Australia) ancestry. In total, 58,648 women (31,716 cases and 26,932 controls) of European ancestry determined by PCs were included in TWAS analyses.

MiXcan, PrediXcan and PredictDB elastic-net mammary tissue models were applied to the individual-level genotype data to perform cell-type-aware or tissue-level TWAS, as described above. All three models were adjusted for the same covariates, including age, country of origin and the top 10 PCs^15^.

#### Evaluation of TWAS findings

We evaluated significant TWAS genes identified by MiXcan and PrediXcan in a substantially larger study of 228,951 European ancestry women (122,977 cases and 105,974 controls) from the combined DRIVE and Breast Cancer Association Consortium (BCAC) GWAS meta-analysis of breast cancer^11^. The summary statistics for the “Combined Oncoarray, iCOGS GWAS meta analysis" were downloaded from https://bcac.ccge.medschl.cam.ac.uk/bcacdata/oncoarray/oncoarray-and-combined-summary-result/gwas-summary-results-breast-cancer-risk-2017/. Associations of predicted tissue-level GReX with breast cancer risk were evaluated using S-PrediXcan^33^ with PredictDB^28^ elastic-net models derived from GTEx v8 mammary tissue data for 337 men and women of European ancestry. We also determined whether TWAS genes identified by MiXcan and PrediXcan were located within 500 kb of 214 previously reported genome-wide significant breast cancer susceptibility loci^11–13^.

### MiXcan software

We developed a computationally efficient R package *MiXcan* to facilitate estimation of cell-type-level GReX prediction models in the two cell components of bulk tissue data, and to perform cell-type-aware TWAS. The *MiXcan* R package, and pre-trained models for the epithelial and stromal (non-epithelial) cell components of mammary tissue derived from 125 European ancestry women in GTEx v8 are freely available at https://github.com/songxiaoyu/MiXcan^54^.

### Reporting summary

Further information on research design is available in the Nature Portfolio Reporting Summary linked to this article.

## Supplementary information

Supplementary Information

Description of Additional Supplementary Files

Supplementary Data 1-2

Reporting Summary

## Data Availability

The data used in this study are publicly available from the following sources: GTEx v8 (dbGaP accession number phs000424.v8.p2 <https://www.ncbi.nlm.nih.gov/projects/gap/cgi-bin/study.cgi?study_id=phs000424.v8.p2>); GTEx v9 (https://gtexportal.org/home/datasets); TCGA (dbGaP accession number phs000178.v8.p7 <https://www.ncbi.nlm.nih.gov/projects/gap/cgi-bin/study.cgi?study_id=phs000178.v8.p7>); Discovery, Biology, and Risk of Inherited Variants in Breast Cancer (DRIVE) (dbGaP accession number phs001265.v1.p1 <https://www.ncbi.nlm.nih.gov/projects/gap/cgi-bin/study.cgi?study_id=phs001265.v1.p1>); PredictDB (http://predictdb.org); and the Breast Cancer Association Consortium (BCAC) (https://bcac.ccge.medschl.cam.ac.uk/bcacdata/). Source data are provided with this paper.

## Source data

Source Data

## Code availability

The *MiXcan* R package is publicly available at https://github.com/songxiaoyu/MiXcan^54^.
