# Supplementary material for: MiXcan: a framework for cell-type-aware transcriptome-wide association studies with an application to breast cancer
Source: Nat Commun. 2023 Jan 23;14:377. doi: 10.1038/s41467-023-35888-4 (PMC9871010; doi:10.1038/s41467-023-35888-4)
Supplement: Supplementary file 1 — Supplementary Information [file 41467_2023_35888_MOESM1_ESM.pdf]

MiXcan: a Framework for Cell-Type-Aware Transcriptome-Wide Association Studies with an Application to Breast Cancer

Song, X. *et al*.

SUPPLEMENTARY FIGURES

Supplementary Figure 1. Comparison of MiXcan proportion estimates with xCell enrichment scores for epithelial cells.

Supplementary Figure 2. Comparison of gene expression prediction accuracy for five methods in an independent validation dataset.

Supplementary Figure 3. Simulation studies to evaluate gene expression prediction accuracy.

Supplementary Figure 4. Simulation studies to evaluate type I error and power by gene expression heritability.

Supplementary Figure 5. Simulation studies to assess cell-type-level associations.

Supplementary Figure 6. Simulation studies to assess the impact of the training data sample size.

Supplementary Figure 7. Transcriptome-wide association studies.

Supplementary Figure 8. Heatmap of mammary tissue gene expression levels.

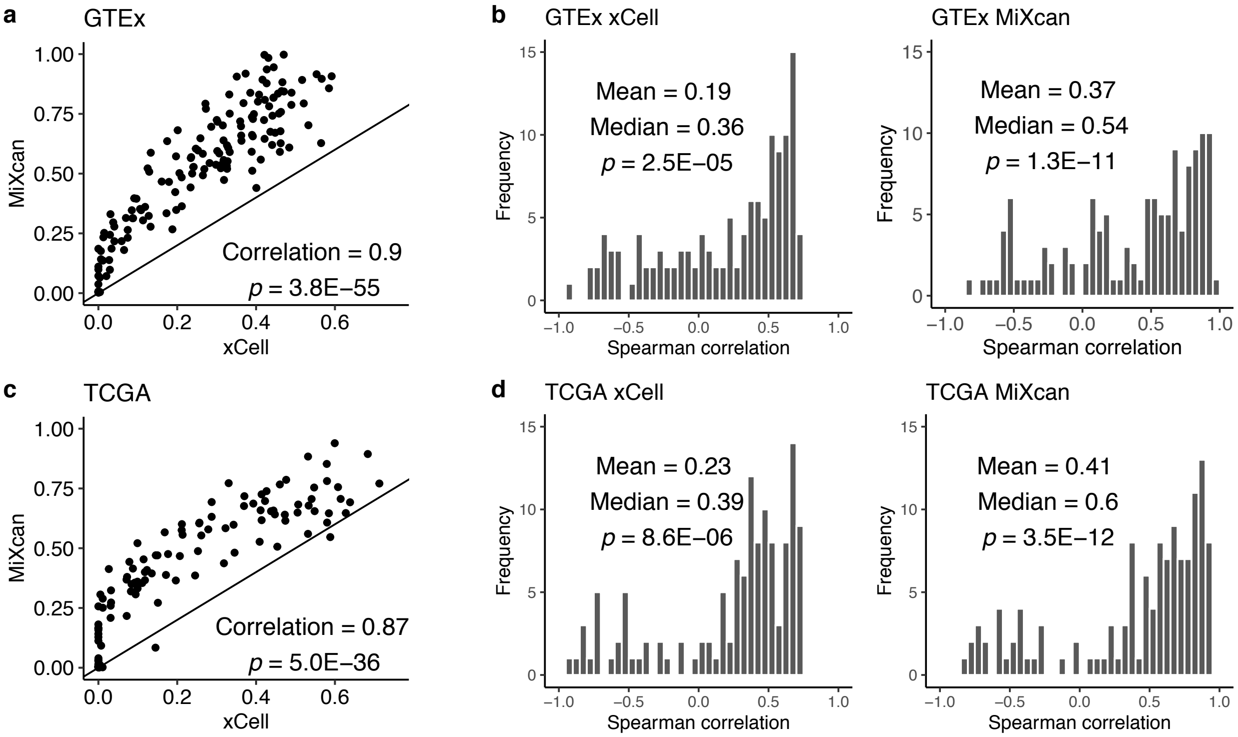

Supplementary Figure 1. Comparison of MiXcan proportion estimates with xCell enrichment scores for epithelial cells. The MiXcan epithelial cell proportion and xCell epithelial cell enrichment score was estimated in normal mammary tissue samples from European ancestry women in GTEx (N=125) and TCGA (N=103). The xCell enrichment score, used as a prior in MiXcan, reflects the relative enrichment for epithelial cells across samples rather than the absolute proportion of epithelial cells within a sample. The MiXcan proportion estimate was highly correlated with the xCell epithelial cell enrichment score in both the GTEx (**a**) and TCGA (**c**) samples, with an estimated correlation of 0.90 (two-sided correlation test p = 3.8E-55) and 0.87 (two-sided correlation test p = 5.0E-36), respectively. Correlations of the measured expression levels for each of 126 genes included in the xCell epithelial cell gene signature tended to be higher with the MiXcan proportion estimate than with the xCell enrichment score in both the GTEx (**b**) and TCGA (**d**) samples. Departures of the distribution of Spearman correlations from zero were evaluated using the two-sided Wilcoxon signed-rank test. Source data are provided as a Source Data file.

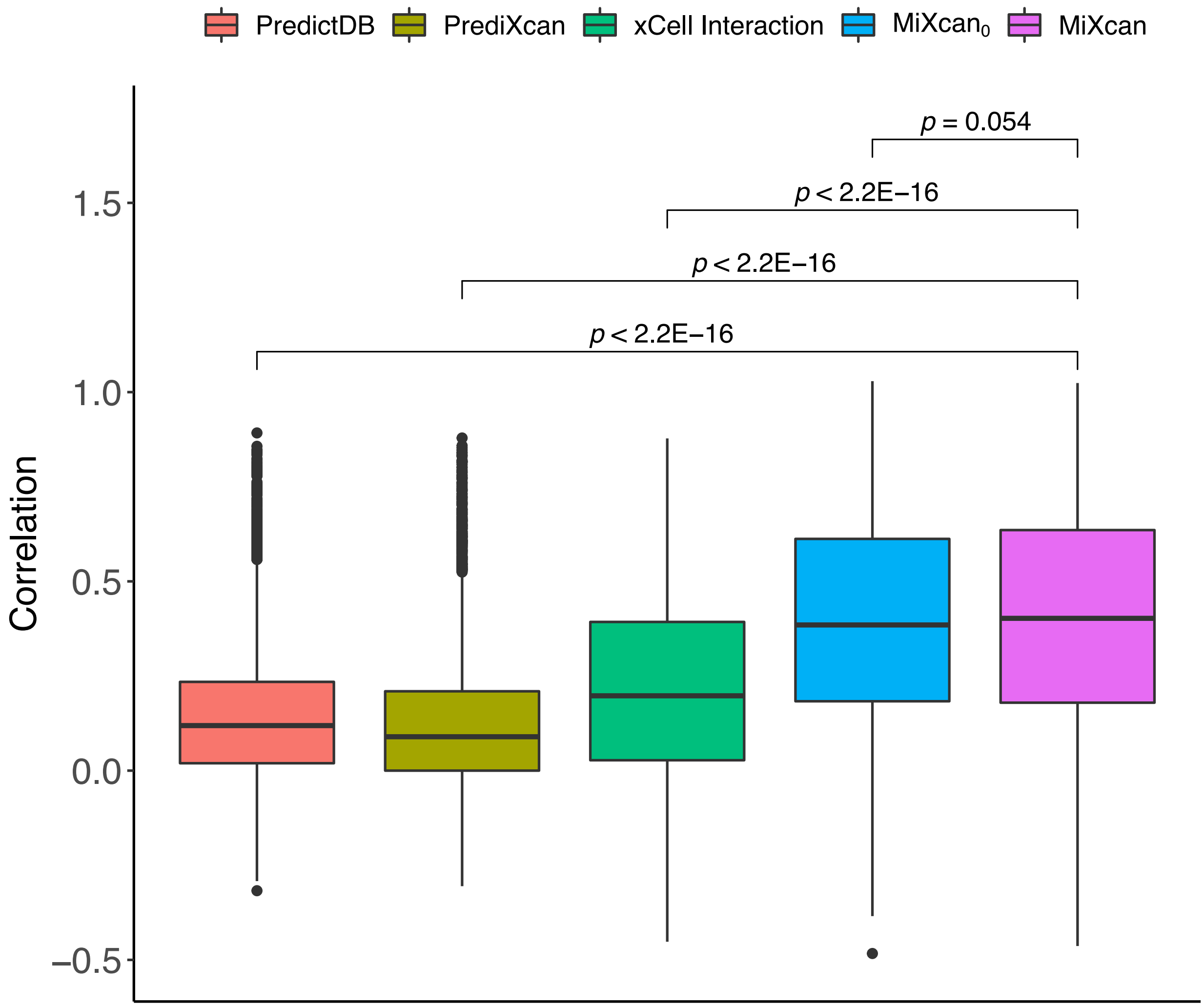

Supplementary Figure 2. Comparison of gene expression prediction accuracy for five methods in an independent validation dataset. Correlations of observed tissue-level gene expression with predicted GReX using five different methods were computed for 6461 genes in an independent dataset of adjacent normal mammary tissue samples from N=103 European ancestry women with breast cancer in TCGA. The publicly available PredictDB (median $r = 0.12$) database includes the tissue-level GReX prediction weights for elastic net models trained using mammary tissue samples from 337 European ancestry men and women in GTEx v8. PrediXcan (median $r = 0.10$) uses the same approach as PredictDB but is trained only on the subset of 125 European ancestry women in GTEx v8. xCell Interaction (median $r = 0.20$) is an elastic net model that includes interactions between genetic variants and the xCell epithelial cell enrichment score. MiXcan_0 (median $r = 0.38$) is an elastic net model that includes interactions between genetic variants and the estimated epithelial cell proportion. Both the xCell Interaction and MiXcan_0 models penalize the two cell-type components asymmetrically and cannot be applied to GWAS datasets without additional transcriptomic or cell-type proportion data. The final MiXcan (median $r = 0.41$) model penalizes the epithelial and stromal cell components symmetrically and can be applied to GWAS datasets. Differences between the correlations obtained for MiXcan versus each of the other methods were compared using the two-sided Wilcoxon signed-rank test. Boxplot bounds show the lower, median, and upper quartiles; whisker lengths are 1.5 times the interquartile range; and points beyond the whiskers are outliers. Source data are provided as a Source Data file.

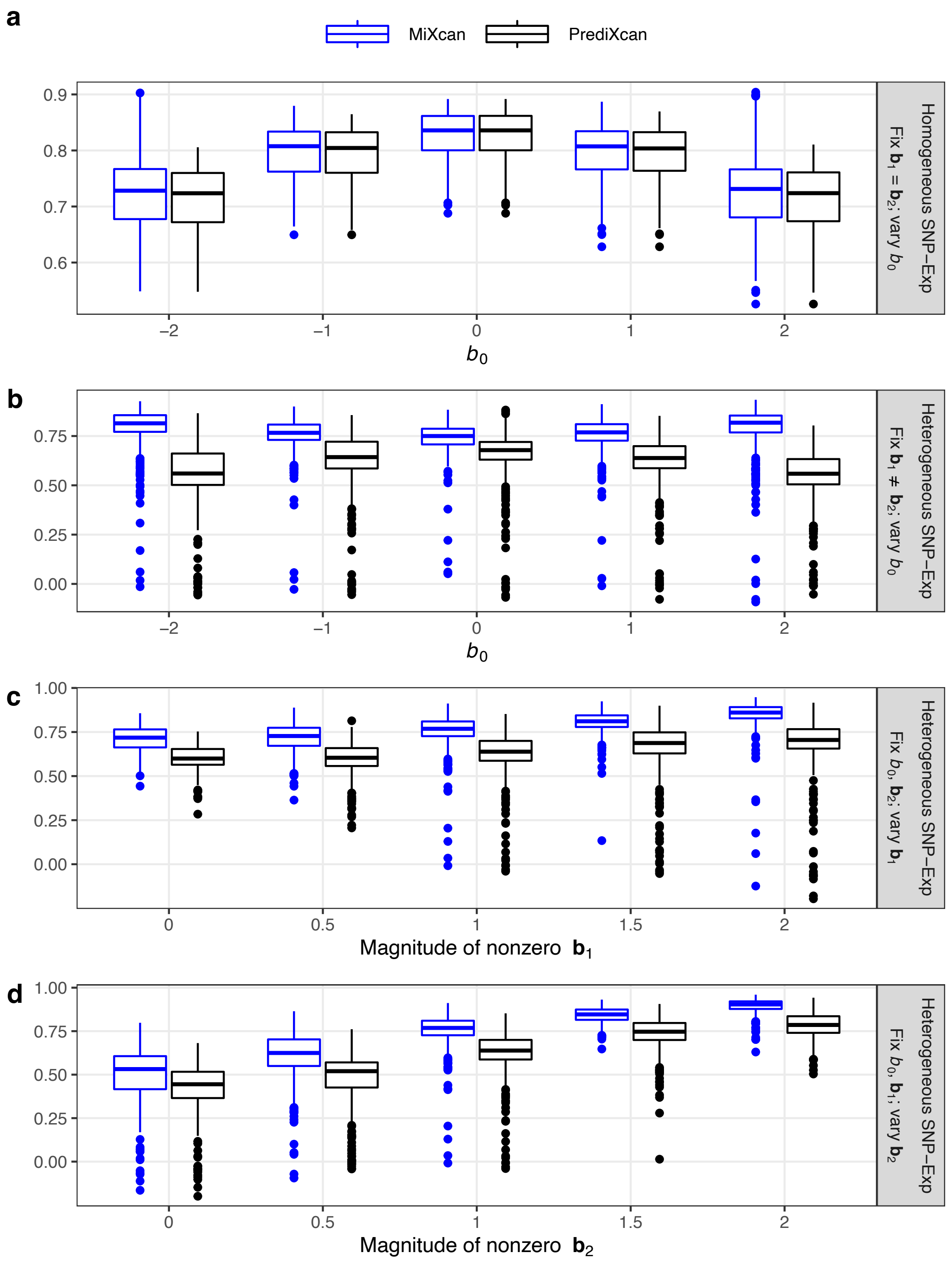

Supplementary Figure 3. Simulation studies to evaluate gene expression prediction accuracy. Correlation of true and predicted tissue-level gene expression using the MiXcan or PrediXcan approaches in independent test sets (N=3000) simulated under a range of realistic data scenarios as shown in Figure 4. **(a)** Homogeneous SNP-Exp associations ($\mathbf{b}_1 = \mathbf{b}_2$) in the two cell types, varying the mean difference in gene expression levels between the two cell types (b_0). Heterogeneous SNP-Exp associations ($\mathbf{b}_1 \neq \mathbf{b}_2$) in the two cell types, varying the: (**b**) mean difference in gene expression levels between the two cell types (b_0); **(c)** magnitude of the SNP-Exp association in the minor cell type ($\mathbf{b}_1$); and (**d**) magnitude of the SNP-Exp association in the major cell type ($\mathbf{b}_2$). Boxplot bounds show the lower, median, and upper quartiles; whisker lengths are 1.5 times the interquartile range; and points beyond the whiskers are outliers. Source data are provided as a Source Data file.

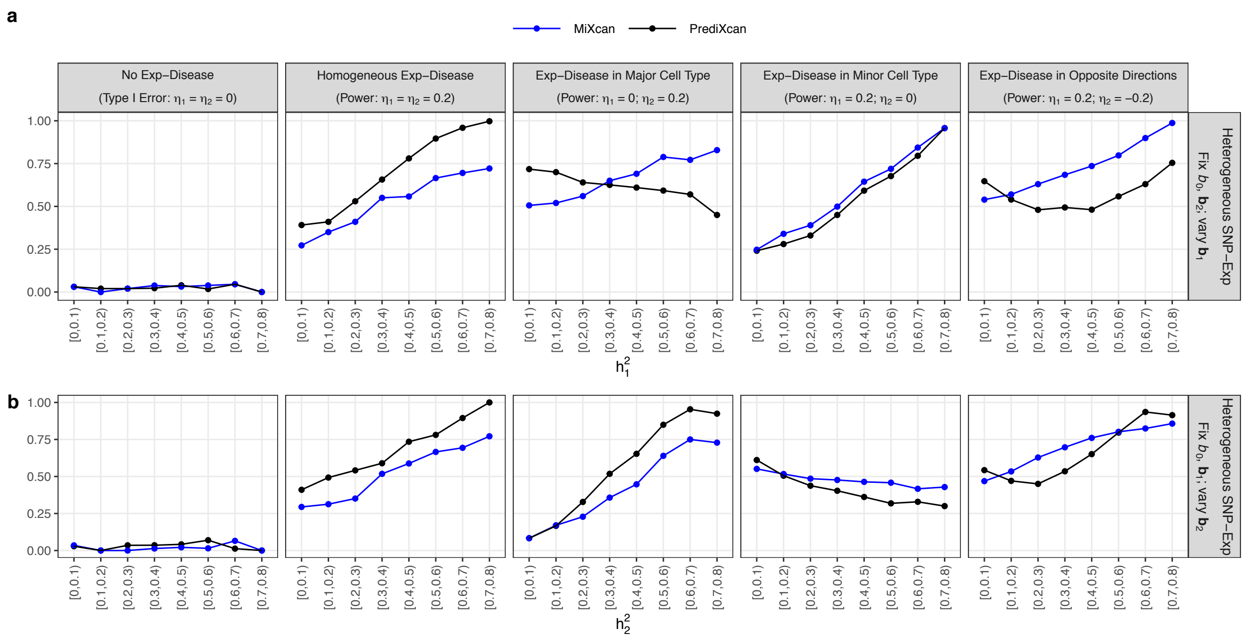

Supplementary Figure 4. Simulation studies to evaluate type I error and power by gene expression heritability. Type I error (column 1) and power (columns 2-5) of MiXcan and PrediXcan according to the gene expression heritability in the (**a**) minor (h_1^2) or (**b**) major (h_2^2) cell type in independent test sets (N=3000) simulated under a range of realistic data scenarios as shown in Figure 4. Source data are provided as a Source Data file.

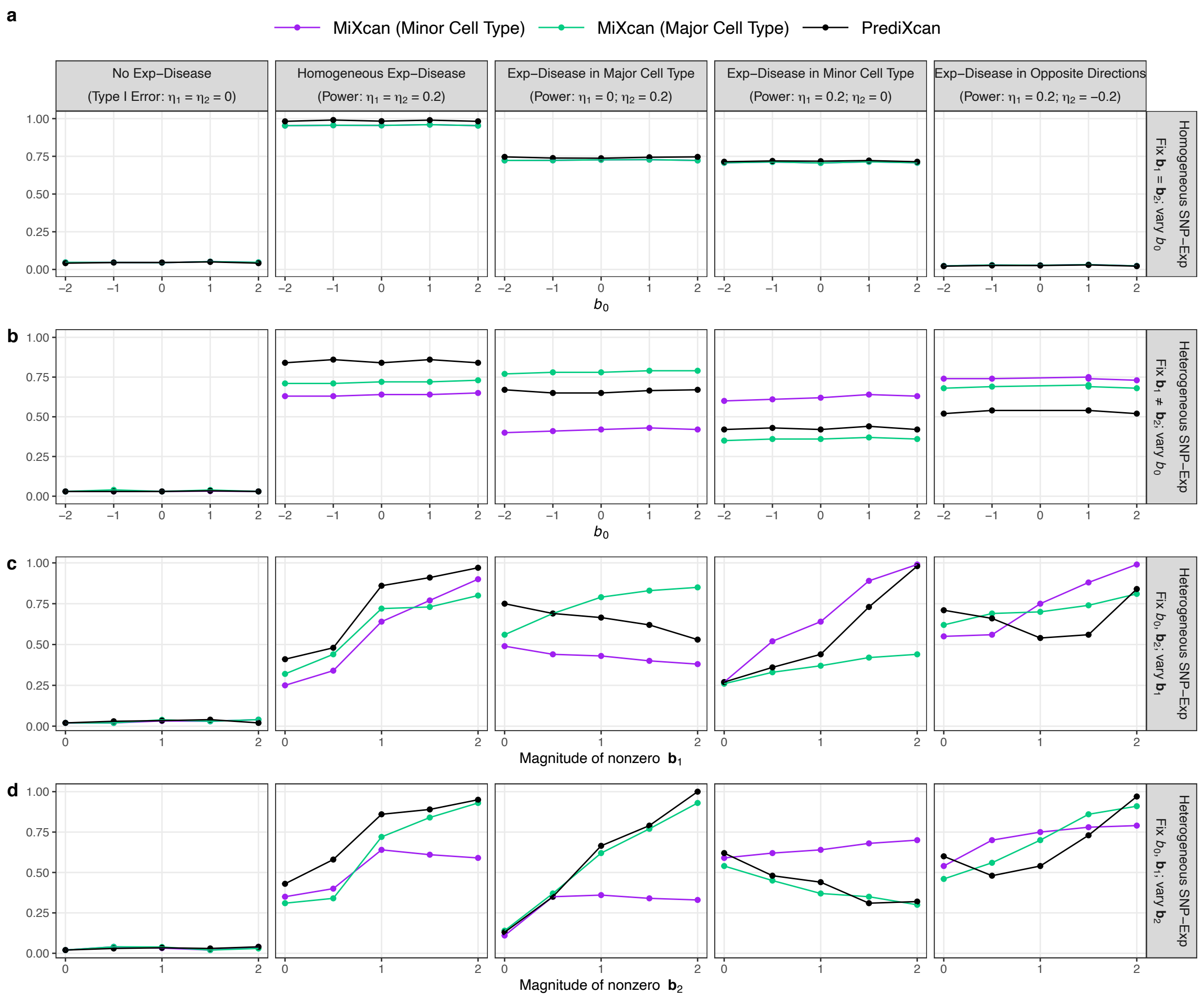

Supplementary Figure 5. Simulation studies to assess cell-type-level associations. Type I error (column 1) and power (columns 2-5) of MiXcan to detect cell-type-level associations of GReX with the disease in the minor and major cell types when the cell-type proportion is accurately estimated. Independent test sets (N=3000) were simulated under a range of realistic data scenarios as shown in Figure 4. **(a)** Homogeneous SNP-Exp associations ($\mathbf{b}_1 = \mathbf{b}_2$) in the two cell types, varying the mean difference in gene expression levels between the two cell types (b_0). Heterogeneous SNP-Exp associations ($\mathbf{b}_1 \neq \mathbf{b}_2$) in the two cell types, varying the: (**b**) mean difference in gene expression levels between the two cell types (b_0); **(c)** magnitude of the SNP-Exp association in the minor cell type ($\mathbf{b}_1$); and (**d**) magnitude of the SNP-Exp association in the major cell type ($\mathbf{b}_2$). Source data are provided as a Source Data file.

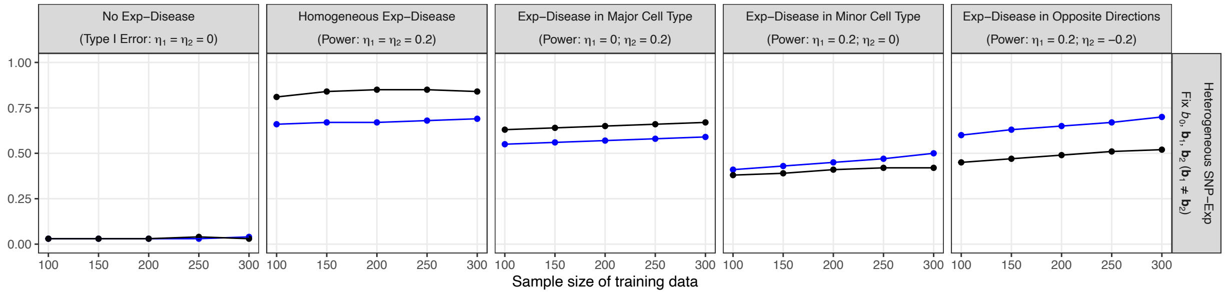

Supplementary Figure 6. Simulation studies to assess the impact of the training data sample size. Type I error (column 1) and power (columns 2-5) of the MiXcan or PrediXcan approaches trained using 100 to 300 samples were evaluated in independent test sets (N=3000) simulated under a range of realistic data scenarios as shown in Figure 4. Source data are provided as a Source Data file.

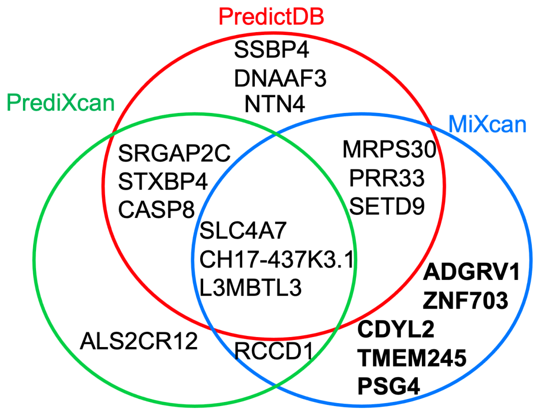

Supplementary Figure 7. Transcriptome-wide association studies. Genes significantly associated with breast cancer using PredictDB, PrediXcan, and MiXcan at $p < 7.7 \times 10^{-6}$ applying a Bonferroni correction for the 6461 genes tested in 31,716 breast cancer cases and 26,932 controls of European ancestry from the DRIVE study. The PredictDB elastic net models were trained using mammary tissue samples from 337 men and women, whereas PrediXcan and MiXcan were trained using only 125 women of European ancestry in GTEx v8. Source data are provided as a Source Data file.

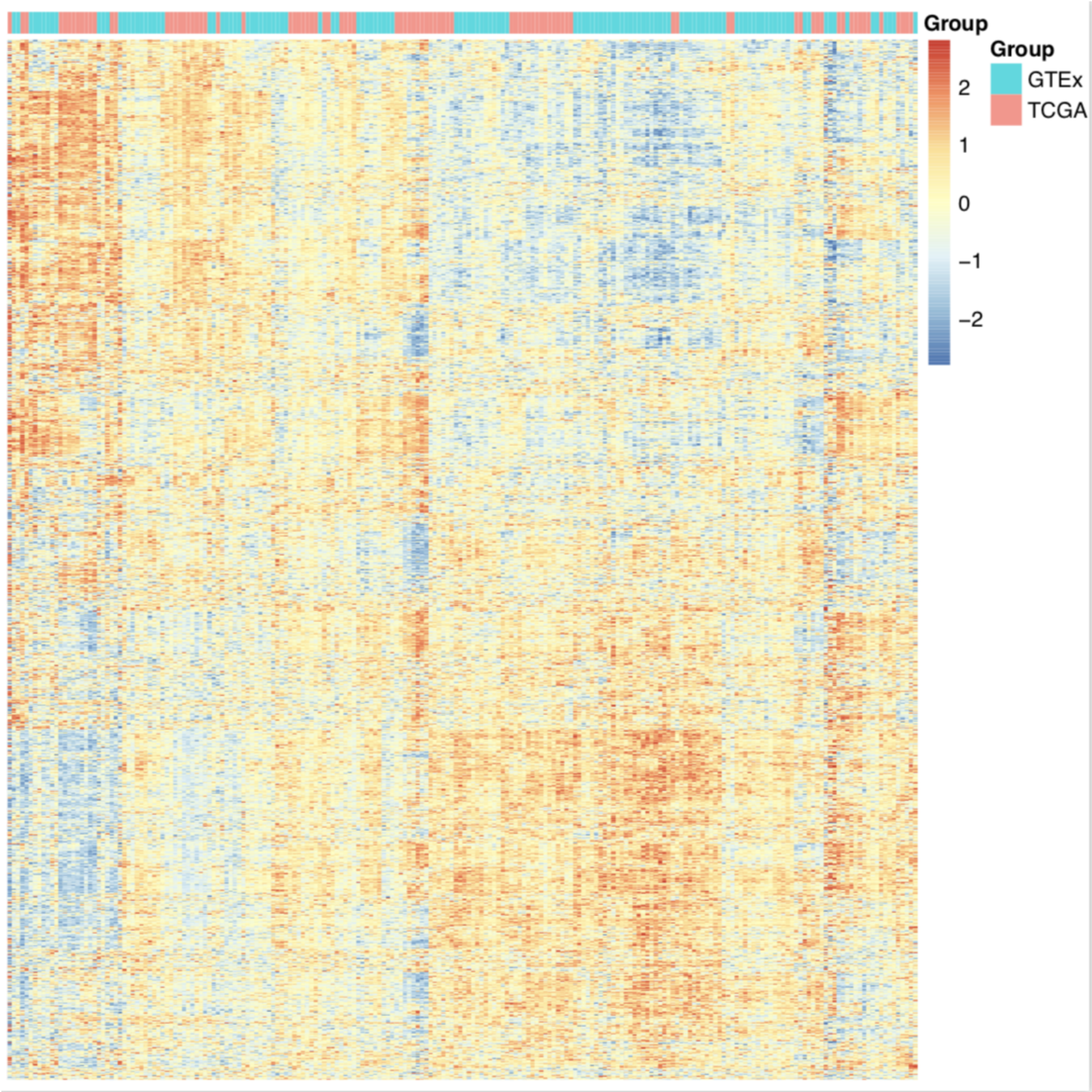

Supplementary Figure 8. Heatmap of mammary tissue gene expression levels. There were no systematic differences between the normalized gene expression levels for 6421 genes present in both the GTEx (N=125) and TCGA (N=103) normal mammary tissue datasets, which were used for training and independent validation, respectively.
