# Supplementary material for: MiXcan: a framework for cell-type-aware transcriptome-wide association studies with an application to breast cancer
Source: Nat Commun. 2023 Jan 23;14:377. doi: 10.1038/s41467-023-35888-4 (PMC9871010; doi:10.1038/s41467-023-35888-4)
Supplement: Supplementary file 2 — Description of Additional Supplementary Files [file 41467_2023_35888_MOESM2_ESM.pdf]

File name: Supplementary Data 1

Description: Genes associated with breast cancer at FDR < 0.10 using the MiXcan or PrediXcan approaches in 58,648 women, and the corresponding S-PrediXcan results in a larger sample of 228,951 women of European ancestry.

File name: Supplementary Data 2

Description: MiXcan GReX prediction models for genes significantly associated with breast cancer risk.
