# Supplementary material for: MiXcan: a framework for cell-type-aware transcriptome-wide association studies with an application to breast cancer
Source: Nat Commun. 2023 Jan 23;14:377. doi: 10.1038/s41467-023-35888-4 (PMC9871010; doi:10.1038/s41467-023-35888-4)
Supplement: Supplementary file 4 — Reporting Summary [file 41467_2023_35888_MOESM4_ESM.pdf]

nature portfolio

Corresponding author(s): Xiaoyu Song, Pei Wang, Weiva Sieh

Last updated by author(s): Nov 29, 2022

Reporting Summary

Nature Portfolio wishes to improve the reproducibility of the work that we publish. This form provides structure for consistency and transparency in reporting. For further information on Nature Portfolio policies, see our Editorial Policies and the Editorial Policy Checklist.

Statistics

For all statistical analyses, confirm that the following items are present in the figure legend, table legend, main text, or Methods section.

n/a	Confirmed	
☐	☒	The exact sample size (*n*) for each experimental group/condition, given as a discrete number and unit of measurement
☐	☒	A statement on whether measurements were taken from distinct samples or whether the same sample was measured repeatedly
☐	☒	The statistical test(s) used AND whether they are one- or two-sided<br>*Only common tests should be described solely by name; describe more complex techniques in the Methods section.*
☐	☒	A description of all covariates tested
☐	☒	A description of any assumptions or corrections, such as tests of normality and adjustment for multiple comparisons
☐	☒	A full description of the statistical parameters including central tendency (e.g. means) or other basic estimates (e.g. regression coefficient) AND variation (e.g. standard deviation) or associated estimates of uncertainty (e.g. confidence intervals)
☐	☒	For null hypothesis testing, the test statistic (e.g. *F*, *t*, *r*) with confidence intervals, effect sizes, degrees of freedom and *P* value noted<br>*Give P values as exact values whenever suitable.*
☒	☐	For Bayesian analysis, information on the choice of priors and Markov chain Monte Carlo settings
☒	☐	For hierarchical and complex designs, identification of the appropriate level for tests and full reporting of outcomes
☐	☒	Estimates of effect sizes (e.g. Cohen's *d*, Pearson's *r*), indicating how they were calculated

Our web collection on statistics for biologists contains articles on many of the points above.

Software and code

Policy information about availability of computer code

Data collection	No software was used.
Data analysis	MiXcan v1.0 <https://github.com/songxiaoyu/MiXcan>, R v4.1.2, edgeR v3.16.5, TOPMed Imputation Server, PLINK v1.90 beta 6.24, EIGENSOFT v6.1.4, BACON v1.26

For manuscripts utilizing custom algorithms or software that are central to the research but not yet described in published literature, software must be made available to editors and reviewers. We strongly encourage code deposition in a community repository (e.g. GitHub). See the Nature Portfolio guidelines for submitting code & software for further information.

Data

Policy information about availability of data

All manuscripts must include a data availability statement. This statement should provide the following information, where applicable:

- Accession codes, unique identifiers, or web links for publicly available datasets
- A description of any restrictions on data availability
- For clinical datasets or third party data, please ensure that the statement adheres to our policy

GTEx v8 data (dbGaP accession number phs000424.v8.p2)
GTEx v9 data (https://gtexportal.org/home/datasets)
TCGA data (dbGaP accession number phs000178.v8.p7)
Discovery, Biology, and Risk of Inherited Variants in Breast Cancer (DRIVE) data (dbGaP accession number phs001265.v1.p1)
PredictDB database GTEx v8 models (http://predictdb.org)

Breast Cancer Association Consortium (BCAC) GWAS summary statistics (https://bcac.ccge.medschl.cam.ac.uk/bcacdata/oncoarray/oncoarray-and-combined-summary-result/gwas-summary-results-breast-cancer-risk-2017/)

Field-specific reporting

Please select the one below that is the best fit for your research. If you are not sure, read the appropriate sections before making your selection.

☒ Life sciences ☐ Behavioural & social sciences ☐ Ecological, evolutionary & environmental sciences

For a reference copy of the document with all sections, see nature.com/documents/nr-reporting-summary-flat.pdf

Life sciences study design

All studies must disclose on these points even when the disclosure is negative.

Sample size	All available mammary tissue samples (N=125) from European ancestry (EA) women in GTEx v8 were used for model development, all available tumor-adjacent normal mammary tissue samples (N=103) from EA women in TCGA were used for model evaluation, and all available genotype data from 58,648 EA women (31,716 cases and 26,932 controls) from the DRIVE breast cancer genome-wide association study (GWAS) were used for transcriptome-wide association study (TWAS) analyses.
Data exclusions	This study included all EA women with data available for analysis.
Replication	The performance of MiXcan models developed using GTEx data was evaluated in an independent dataset from the TCGA. In TWAS analyses, statistical significance was determined using a Bonferroni correction for the number of genes tested. Significant genes were further evaluated in a larger cohort of 228,951 EA women (122,977 cases and 105,974 controls) using BCAC GWAS summary statistics and conventional TWAS models for mammary tissue-level gene expression.
Randomization	Not applicable. This study did not involve experimental group allocation.
Blinding	Not applicable. This study did not involve experimental group allocation.

Reporting for specific materials, systems and methods

We require information from authors about some types of materials, experimental systems and methods used in many studies. Here, indicate whether each material, system or method listed is relevant to your study. If you are not sure if a list item applies to your research, read the appropriate section before selecting a response.

Materials & experimental systems

n/a	Involved in the study	
☒	☐	Antibodies
☒	☐	Eukaryotic cell lines
☒	☐	Palaeontology and archaeology
☒	☐	Animals and other organisms
☒	☐	Human research participants
☒	☐	Clinical data
☒	☐	Dual use research of concern

Methods

n/a	Involved in the study	
☒	☐	ChIP-seq
☒	☐	Flow cytometry
☒	☐	MRI-based neuroimaging
